# Origin and diversification of Lake Ohrid’s endemic acroloxid limpets: the role of geography and ecology

**DOI:** 10.1186/s12862-016-0826-6

**Published:** 2016-12-15

**Authors:** Björn Stelbrink, Alena A. Shirokaya, Kirstin Föller, Thomas Wilke, Christian Albrecht

**Affiliations:** 1 Department of Animal Ecology and Systematics, Justus Liebig University Giessen, Heinrich-Buff-Ring 26-32, 35392 Giessen, Germany; 2 Limnological Institute, Siberian Branch of Russian Academy of Sciences, Ulan-Batorskaya Str., 3, P.O. Box 4199, 664033 Irkutsk, Russia

**Keywords:** Freshwater limpets, Ancient lakes, Balkans, Molecular phylogeny, Molecular clock, Biogeography, Phylogeography, Incipient speciation

## Abstract

**Background:**

Ancient Lake Ohrid, located on the Albania-Macedonia border, is the most biodiverse freshwater lake in Europe. However, the processes that gave rise to its extraordinary endemic biodiversity, particularly in the species-rich gastropods, are still poorly understood. A suitable model taxon to study speciation processes in Lake Ohrid is the pulmonate snail genus *Acroloxus*, which comprises two morphologically distinct and ecologically (vertically) separated endemic species. Using a multilocus phylogenetic framework of *Acroloxus* limpets from the Euro-Mediterranean subregion, together with molecular-clock and phylogeographic analyses of Ohrid taxa, we aimed to infer their geographic origin and the timing of colonization as well as the role of geography and ecology in intra-lacustrine diversification.

**Results:**

In contrast to most other endemic invertebrate groups in Lake Ohrid, the phylogenetic relationships of the endemic Ohrid *Acroloxus* species indicate that the Balkan region probably did not serve as their ancestral area. The inferred monophyly and estimated divergence times further suggest that these freshwater limpets colonized the lake only once and that the onset of intra-lacustrine diversification coincides with the time when the lake reached deep-water conditions ca 1.3 Mya. However, the difference in vertical distribution of these two ecologically distinct species is not reflected in the phylogeographic pattern observed. Instead, western and eastern populations are genetically more distinct, suggesting a horizontal structure.

**Conclusions:**

We conclude that both geography and ecology have played a role in the intra-lacustrine speciation process. Given the distinct morphology (sculptured vs. smooth shell) and ecology (littoral vs. sublittoral), and the timing of intra-lacustrine diversification inferred, we propose that the onset of deep-water conditions initially triggered ecological speciation. Subsequent geographic processes then gave rise to the phylogeographic patterns observed today. However, the generally weak genetic differentiation observed suggests incipient speciation, which might be explained by the comparatively young age of the lake system and thus the relatively recent onset of intra-lacustrine diversification.

**Electronic supplementary material:**

The online version of this article (doi:10.1186/s12862-016-0826-6) contains supplementary material, which is available to authorized users.

## Background

Ancient lakes are famous hotspots of biodiversity and represent natural laboratories to study evolution [1–6]. These extant long-lived lakes, which have continuously existed for more than 100,000–500,000 years (see [7–9]), can act as evolutionary reservoirs. At the same time, species may evolve through intra-lacustrine speciation (‘cradle function’; e.g., [3, 10, 11]). Comparatively little is known about the European ancient lakes, partly because it is still unclear which European lacustrine systems qualify as ancient lakes. Undisputedly ‘ancient’ is the oligotrophic and karstic Balkan Lake Ohrid (Macedonia/Albania), a steep-sided graben lake with tectonic origin. Lake Ohrid is situated 693 m above sea level and has a maximum length of 30.4 km and a maximum width of 14.8 km. It has a mean depth of 155 m and a maximum depth of 293 m [12]. The lake is mainly fed by springs and precipitation, and drains into the northern Crni Drim River, which belongs to the Adriatic drainage system.

The age of Lake Ohrid has been highly debated and estimates mainly based on biological data suggest a maximum age of 2–3 My [9, 13]. However, seismological and sedimentological data obtained in the course of the SCOPSCO (Scientific Collaboration On Past Speciation Conditions in Lake Ohrid) deep-drilling program, conducted in spring 2013, revealed an age of at least 1.3 My for deep-water conditions [14] and c. 2.0 My for its oldest sediments [12].

Although it is clear that Lake Ohrid is home to a disproportional large number of gastropod endemics (74 gastropod species, 56 of which are endemic; see [13, 15, 16]), the evolutionary history and processes leading to these unique faunas are still largely unknown. However, recent studies provided insights into the geographic origin of various groups (e.g., Balkan vs. non-Balkan affinities; see below), revealed constant rates of diversification in hydrobiid snails [16], and identified different metacommunity processes (e.g., dispersal limitation, species interaction) promoting geographic and ecological speciation in gastropods [17].

The Acroloxidae is one of the four freshwater pulmonate families (besides Lymnaeidae, Physidae, and Planorbidae) inhabiting Lake Ohrid and represents a well-defined monophyletic group that is characterized by several distinct morphological and anatomical features and is further supported by molecular studies [18–26]. This family shows a Holarctic distribution pattern with two widespread species recognized, *Acroloxus lacustris* from Europe [27–29] and *A. coloradensis* from North America [30–32]. There are also numerous point endemics such as *A. tetensi* from Cave Planinska jama (Slovenia) and *A. egirdirensis* from ancient Lake Eǧirdir (Turkey). However, relatively little is known about interspecific relationships in the Acroloxidae (but see [33] for a preliminary Euro-Mediterranean phylogeny including *A. egirdirensis* and some *A. lacustris* populations and [34] for population genetics on few *A. coloradensis* populations, respectively). Only recently, phylogenetic relationships have been studied in Lake Baikal’s endemic acroloxid species flock (25 species) based on molecular markers, revealing that intra-lacustrine speciation during the Plio-Pleistocene gave rise to several littoral, sublittoral, and even abyssal species inhabiting oil-seeps and hydrothermal vents below 100 m water depth. However, a reasonable colonization scenario (littoral-abyssal or abyssal-littoral) could not be inferred from the phylogenetic relationships and the vertical distribution pattern of the endemic species [35].

In Lake Ohrid, the widespread European *A. lacustris* (however, only found in low numbers) and at least two endemic species co-occur, namely *A. macedonicus* and *A. improvisus* [15, 36, 37]. The latter two distinctly differ in their shell morphology (sculptured vs. smooth shell) and some anatomical characters [36]. Such a shell sculpturing is a very rare phenomenon among freshwater pulmonates and can only be observed in a few taxa inhabiting ancient lakes (e.g., *Ancylus* in Lake Ohrid, [38]; *Baicalancylus* and *Gyraulus* (*Armiger*) in Lake Baikal, [39–41]; *Protancylus* in the Malili lakes and Lake Poso, [42]) and apparently represents a case of shell convergence. Shell sculpturing as displayed by *A. macedonicus* could be either related to an increased wave activity in the littoral [36] or may act as a defence strategy [38]. From an ecological perspective, the two endemic species seem to live under strict allopatric conditions, related to different habitats occupied (vertical separation). In general, both limpet species are only found on hard substrate in Lake Ohrid. However, while *A. improvisus* is mainly found on bivalve shells of *Dreissena polymorpha* in the upper sublittoral between 18 and 35 m, *A. macedonicus* inhabits the upper littoral (0–0.5 m water depth) and occurs on rocks and limestone boulders, [36, 37, 43]. On a horizontal scale, Hubendick [36, 43] suggested the existence of two morphologically and anatomically different *A. macedonicus* populations that are geographically isolated, one inhabiting the rocky littoral in the east, the second occurring in the north-eastern part of the lake.

Here, we examine patterns of speciation as well as the underlying evolutionary processes in freshwater acroloxid limpets across their native range in Europe with particular focus on Lake Ohrid using a combination of mitochondrial and nuclear markers. Specifically, we aim to 1) infer the geographic origin of Lake Ohrid acroloxid endemics using molecular phylogenetic analyses, 2) infer the timing of intra-lacustrine speciation events using molecular-clock analyses, and 3) study the genetic differentiation among various populations across the lake and across potential ecological and geographic clines using phylogeographic analyses.

## Methods

### Substrate-type distribution analysis

**Fig. 1:**
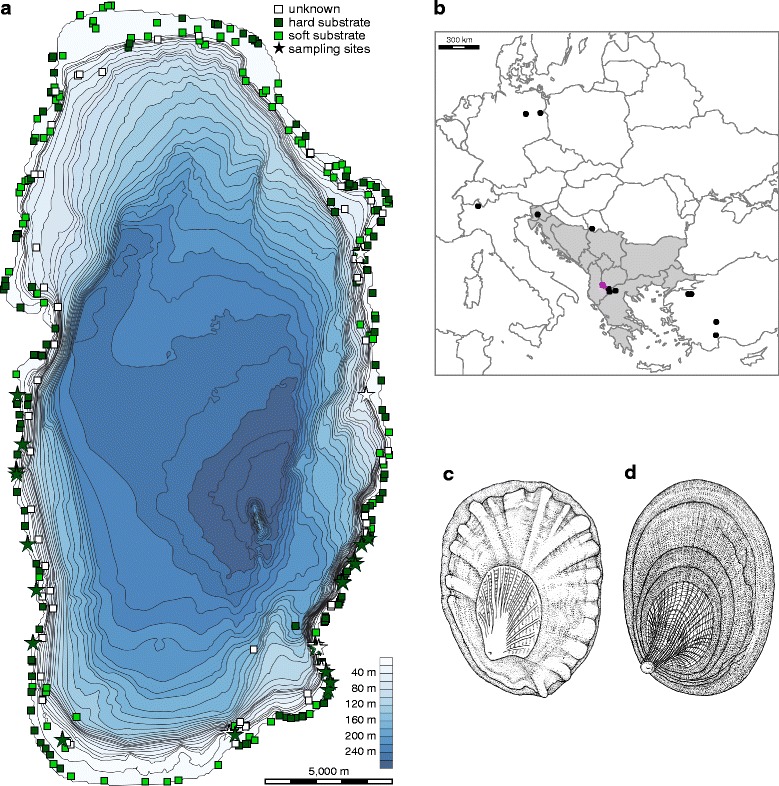
Substrate types and sampling sites inside and outside Lake Ohrid. **a** Bathymetric map of Lake Ohrid with 10 m contour lines. Coloured rectangles represent substrate types for a particular locality classified based on information recorded during field trips (see Methods for details on substrate classification). Sampling sites of *Acroloxus* are colour-coded according to substrate type, **b** Map of the Euro-Mediterranean subregion with sampling sites (grey: Balkans; pink: Lake Ohrid; © d-maps.com), **c** Shell of the littoral *A. macedonicus*, **d** Shell of the sublittoral *A. improvisus*

Substrate-type distribution within Lake Ohrid was indirectly reconstructed in order to estimate the potential impact of substrate on species distribution by using recorded data from a total of 364 localities sampled in the lake between 2003 and 2013 (Fig. 1a). Substrate was classified into three types: 0 – unknown (*n* = 71, c. 19.5%), 1 – mainly hard substrate (*n* = 171, c. 47.0%; rocks: >200 mm, stones: 63–200 mm, gravel: 2–63 mm), and 2 – mainly soft substrate (*n* = 122, 33.5%; sand: 0.063–2 mm, silt: <0.063 mm, sapropel, detritus). Sampling sites and substrate-specific localities were plotted on a bathymetric map showing 10 m contour lines using QGIS v. 2.10.1 [44].

### Taxon sampling, DNA extraction, amplification and sequencing

Material was mainly collected from Lake Ohrid, the Balkans and other localities across the Euro-Mediterranean subregion (Fig. 1). Individuals were obtained by hand-collecting from hard substrate in shallow waters or from stones and rocks lifted from depths up to 5 m by snorkelling. Deeper parts of the littoral and sublittoral up to 60 m were sampled using a dredge. Ohrid specimens were identified based on shell morphology and bathymetrical zonation (littoral = *A. macedonicus* vs. sublittoral = *A. improvisus*) suggested by Hubendick [36]. DNA of 86 specimens representing 5 described species (all 4 European species plus *A. egirdirensis*) and a single undescribed population from the Anatolian Lake Kırkgöz were isolated using the protocol of Winnepenninckx et al. [45]. Two mitochondrial (COI and 16S rRNA) and three nuclear loci (28S rRNA, H3, and ITS2) were amplified using the following PCR conditions: 95 °C – 1 min; 35 cycles: 95 °C – 30 s, 52 °C – 30 s, 72 °C – 30 s; final elongation at 72 °C – 3 min; see Table 1 for a list of primers) and visualized on either a Long Read IR2 4200 sequencer (LICOR, Lincoln, NE, USA) using the Thermo Sequenase fluorescent labelled primer cycle sequencing kit (Amersham Pharmacia Biotech, Piscataway, NJ, USA) or an ABI 3730 XL sequencer (Life Technologies) using a Big Dye Terminator Kit (Life Technologies). Sequences are deposited in GenBank, accession numbers KY092673-KY092894 (Additional file 1: Table S1).

**Table 1:** Primers used for sequencing

Primer	5′–3′ sequence	Source
16Sar	CGC CTG TTT ATC AAA AAC AT	[92]
16Sbr	CCG GTC TGA ACT CAG ATC ACG T	[92]
28SD23F	GAG AGT TCA AGA GTA CGT G	[93]
28SD6R	CCA GCT ATC CTG AGG GAA ACT TCG	[93]
LCO1490	GGT CAA CAA ATC ATA AAG ATA TTG G	[94]
COR722b	TAA ACT TCA GGG TGA CCA AAA AAT YA	[95]
H3F	ATG GCT CGT ACC AAG CAG ACV GC	[96]
H3R	ATA TCC TTR GGC ATR ATR GTG AC	[96]
LT1 (ITS2)	TCG TCT GTG TGA GGG TCG	[97]
ITS2-RIXO	TTC TAT GCT TAA ATT CAG GGG	[98]

### Phylogenetic analyses

Twenty-five European and Anatolian *Acroloxus* specimens, seven representatives of Ohrid endemics (two *A. macedonicus*, three non-ribbed *A. macedonicus*, and two *A. improvisus* individuals) and three outgroup taxa, representing Latiidae (*Latia neritoides*), Lymnaeidae (*Lymnaea stagnalis*) and Planorbidae (*Planorbarius corneus*), were included in the phylogenetic analyses, for which all four markers were available, except for the single GenBank specimen (see Additional file 1: Table S1 for a detailed list of specimens examined). 16S and 28S rRNA sequences were aligned using the MAFFT web service [46]. Together with the protein-coding COI and H3 datasets, they resulted in a final alignment of 2,208 bp (35 sequences. Genetic variation was comparatively low in the two nuclear markers, revealing 18 variable sites (3 for the Ohrid endemics) in 28S rRNA and 6 variable sites (1 in the Ohrid group) in H3. Different genes were treated as single partitions in all subsequent analyses. PartitionFinder 1.1.1 for Windows [47] was used in order to test for subset partitions (settings: all models, AIC, codon partitions not used, greedy). The best partition scheme suggested four partitions according to the genetic markers used. Best-fit substitution models were selected for each partition for the criteria AIC and AICc using jModelTest v. 0.1.1 ([48]; see Table 2). Phylogenetic analyses were conducted using RAxML BlackBox [49] with the GTR + Γ model for each of the four partitions as implemented in RAxML, and MrBayes 3.1.2 [50] using the substitution models selected for the AIC and AICc according to jModelTest and the following parameters: ngen = 1,000,000, samplefreq = 50, burn-in = 10,001.

**Table 2:** Best-fit substitution models for the different partitions estimated with jModelTest

Partition	Length (bp)	AIC	AICc
16S rRNA	468	GTR + Γ	GTR + Γ
28S rRNA	757	GTR + Γ	GTR + Γ
COI	655	HKY + Γ	HKY + Γ
H3	328	GTR + I	GTR + I

### Estimation of divergence times

Estimation of divergence times was performed in BEAST v. 1.8.0 [51] using different clock models and tree prior (STR-BD: strict clock, birth-death process; STR-Y: strict clock, Yule process; UCLN-BD: uncorrelated lognormal relaxed clock, birth-death process; and UCLN-Y: uncorrelated lognormal relaxed clock, Yule process), and running four replicates on the CIPRES Science Gateway web portal [52] with the following settings: ngen = 100,000,000; samplefreq = 5,000; burn-in = 10,001. A first run for the UCLN-Y resulted in low ESS values for the prior and posterior distribution. Therefore, the less complex HKY substitution model was applied to the 16S rRNA, 28S rRNA and H3 partitions in each of the four analyses (see e.g., [53, 54]). A mean molecular clock rate (uniform prior) ranging from 0.0124 to 0.0157 (substitutions per site and My) proposed for the COI gene for different Protostomia groups referring to the substitution models HKY and HKY + I + Γ, respectively was used (see [55]). Log and tree files of replicates were combined in LogCombiner v. 1.8.0 (BEAST package; 75% burn-in) after checking the replicates for congruency in Tracer v. 1.5 [56]. The four final log files were subjected to a Bayes factor (BF) analysis as implemented in Tracer v. 1.5 comparing the tree likelihood with 1,000 bootstrap replicates. MCC files were selected and annotated in TreeAnnotator v. 1.8.0 (BEAST package; no additional burn-in) summarizing the entire posterior distribution including a total of 20,000 trees. As mitochondrial markers such as 16S rRNA and COI are genetically linked, we performed additional species tree analyses in *BEAST (implemented in the BEAST package; [57]). Species were defined as follows (and thus refer to reciprocally monophyletic clades revealed by previous phylogenetic analyses): central European *A. lacustris*, *A. tetensi*, *Acroloxus* sp. (Lake Kırkgöz), *A. egirdirensis*, *Acroloxus* sp. (Lake Mergozzo), and Lake Ohrid endemics. 16S rRNA and COI were linked in the tree model (ploidy type: mitochondrial); the two nuclear markers (28S rRNA and H3; ploidy type: autosomal nuclear) were treated as independent markers. Substitution models, clock models and MCMC settings were the same as in the BEAST analyses (species tree priors: Yule and birth-death process; population size model: piecewise linear & constant root). Files were combined and tested using Bayes factors as described above.

### Phylogeographic analyses

Haplotype networks were generated for the mitochondrial COI and 16S rRNA (plus a combined network) and the nuclear ITS2 datasets using TCS v. 1.2.1 [58] with a default connection limit of 95% (gaps treated as fifth state) for all endemic Ohrid populations collected from a total of 27 localities. The COI dataset included 61 specimens (*A. improvisus*: *n* = 21; *A. macedonicus*: *n* = 40), while only a reduced number of specimens was sequenced for the 16S rRNA (total: *n* = 26; *A. improvisus*: *n* = 11; *A. macedonicus*: *n* = 15) and ITS2 datasets (total: *n* = 30; *A. improvisus*: *n* = 17; *A. macedonicus*: *n* = 13). Because 28S rRNA and H3 showed only little genetic variation for the Ohrid endemics (3 and 1 variable sites, respectively), these markers were not used for such networks. Genetic distances (uncorrected p-distances) for COI were calculated in MEGA v. 6.06 [59] for the Lake Ohrid endemics.

## Results

### Substrate type distribution across Lake Ohrid

The analysis of substrate types revealed a non-homogenous distribution of hard and soft substrates across the lake. Suitable hard substrates for freshwater limpets and other rock-dwelling mollusc species are particularly found along the western and eastern shorelines. These hard-substrate habitats are horizontally separated by long stretches of mainly soft substrate (mud, sand) in the northern and southern parts of the lake (Fig. 1). Accordingly, freshwater limpets were only found along the western and eastern shore, with the highest abundance (and genetic diversity) observed in the south-eastern part of the lake (Fig. 1). Populations from the very north-eastern part of the lake, as reported by Hubendick [36, 43] (see Introduction), have not been found during our surveys.

### Phylogenetic relationships and spatiotemporal patterns

**Fig. 2:**
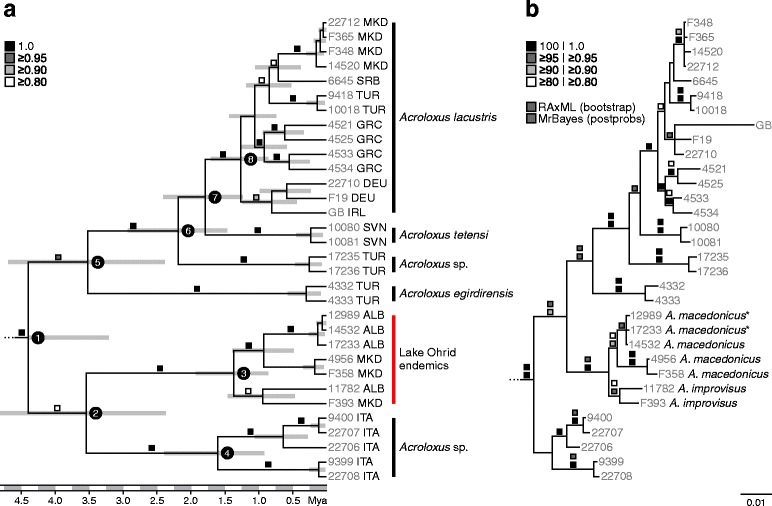
Phylogenetic relationships and estimation of divergence times of Euro-Mediterranean *Acroloxus* species and populations. **a** BEAST MCC tree (UCLN-Y) based on 16S rRNA, 28S rRNA, COI and H3 with selected node ages (see Table 4), posterior probabilities and 95% HPD (outgroup removed). Country codes used: ALB = Albania, DEU = Germany, GRC = Greece, IRL = Ireland (GenBank sequence, GB), ITA = Italy, MKD = Macedonia, SVN = Slovenia, SRB = Serbia, and TUR = Turkey, **b** MrBayes phylogram with posterior probabilities and RAxML bootstrap values (outgroup removed). Acroloxus macedonicus* individuals refer to non-ribbed specimens of *A. macedonicus*

The phylogenetic analyses revealed a nearly congruent topological pattern for the species and populations studied (Fig. 2). Most interestingly, the Lake Ohrid endemics are neither closely related to *A. lacustris* from the Balkan lakes Prespa, Mikri Prespa, and Vegoritida, nor to *A. lacustris* sampled in the vicinity of Lake Ohrid. The latter two groups are nested within the widespread central European *A. lacustris* clade (Fig. 2). Moreover, several highly supported reciprocally monophyletic groups could be identified including a heterogeneous *A. lacustris* clade containing populations from central and western Europe, the Balkans (including Greece and Macedonia) and Anatolia (Lake Uluabat). Potential sister to this clade is the endemic cave-dwelling *Acroloxus tetensi.* However, support values are comparatively low. Further highly supported groups are the endemic *A. egirdirensis* (Lake Eǧirdir) and a population from the Anatolian Lake Kırkgöz. Interestingly, the populations sampled from the North Italian Lake Mergozzo are genetically different from the remaining central and western European *A. lacustris* populations. However, given the slightly different topologies among the analyses and the comparatively low support values in the BEAST analysis, it remains unclear whether these populations from Lake Mergozzo represent the sister group to the Lake Ohrid endemics or whether this group is sister to the remaining European/Anatolian species and populations.

For the molecular clock analyses, the ESS values visualized in Tracer v. 1.5 were considerably higher than 200 in each of the 4 analyses. The BF analysis only slightly favoured the UCLN-Y model compared to the UCLN-BD model, but showed a decisive support for the UCLN-Y model compared to both strict-clock analyses (see Table 3; positive support: 0–3; strong support: 3–6; decisive support for null hypothesis: >6; see e.g., [60]). For the *BEAST analyses, the BF analysis favoured the *UCLN-BD model over the *UCLN-Y model and the two strict-clock models (Table 3). The application of different tree and clock priors mainly affected divergence times at the basal nodes, while the effect on the ingroup and further internal nodes was less pronounced (Table 4). Moreover, the topologies remained congruent among the analyses except for the position of *A. lacustris* from Ireland (Additional file 2: Figure S1). Age estimates based on the favoured UCLN-Y analysis suggest that the MRCA (most recent common ancestor) of *Acroloxus* originated c. 4.44 (mean; 95% HPD, highest posterior density interval: 3.23, 5.76) Mya and thus between the very late Miocene and early Pliocene (node 1 in Fig. 2a; see Table 4). Divergence times between *Acroloxus* and the closest outgroup taxon (*Planorbarius corneus*) in the BEAST analysis are estimated to be c. 14.27 (8.70, 19.97) My. However, age estimates derived for this split and the root height should be considered with caution because diversification within this timeframe (>10 My) may be affected with substitution saturation, particularly for the mitochondrial marker COI (see e.g., [55]). The age of the potential split between the focal Lake Ohrid group and the Lake Mergozzo clade is estimated to c. 3.58 (2.39, 4.85) My and thus dates back to the Pliocene, while the MRCA of the Lake Ohrid endemics is considerably younger with an estimated age of c. 1.37 (0.86, 1.94) My (nodes 2 and 3). The split between *A. egirdirensis* and the remaining populations (node 5) have occurred at approximately the same time as the split between Lake Mergozzo and Lake Ohrid. Further internal nodes of interest all date back to the Pleistocene with the youngest clade of interest, comprising the widespread *A. lacustris,* being c. 1.27 (0.86, 1.72) My old (node 8). Divergence time estimates obtained from the *BEAST analyses are very similar to the node ages estimated by the previous BEAST analyses (see Table 4 for the favoured *UCLN-BD model and Additional file 2: Figure S2 for the four species trees including both mean ages and posterior probabilities). The only considerable difference is found among the most internal nodes of interest (nodes 5–7), which show slightly younger mean ages compared to the BEAST analyses, in which the mitochondrial markers were unlinked and a single tree model was applied to the four genetic markers (partitions) used.

**Table 3:** Results of the BF analysis (log_10_ Bayes factors)

	Ln P (model | data)	S.E.	STR-BD/*STR-BD	STR-Y/*STR-Y	UCLN-BD/*UCLN-BD	UCLN-Y/*UCLN-Y
STR-BD	−8,802.908	+/− 0.126	-	2.032	−46.650	−46.962
STR-Y	−8,807.588	+/− 0.163	−2.032	-	−48.683	−48.995
UCLN-BD	−8,695.492	+/− 0.269	46.650	48.683	-	−0.312
UCLN-Y	−8,694.774	+/− 0.267	46.962	48.995	0.312	-
*STR-BD	−8704.524	+/− 0.238	-	−0.002	−26.918	−26.844
*STR-Y	−8704.518	+/− 0.245	0.002	-	−26.915	−26.842
*UCLN-BD	−8642.544	+/− 0.256	26.918	26.915	-	0.073
*UCLN-Y	−8642.712	+/− 0.286	26.844	26.842	−0.073	-

The favoured analyses are UCLN-Y and *UCLN-BD (models marked with an asterisk refer to the *BEAST species tree analyses). See Methods for details

**Table 4:** Estimated divergence times in My obtained for the four molecular-clock analyses

	STR-BD	STR-Y	UCLN-BD	UCLN-Y	*UCLN-BD
RootHeight	42.45 (31.37, 55.11)	35.91 (26.42, 45.97)	31.71 (18.32, 45.95)	20.63 (13.24, 28.79)	34.23 (21.08, 52.34)
Node 1	3.86 (2.98, 4.78)	3.82 (2.99, 4.78)	4.49 (3.23, 5.87)	4.44 (3.23, 5.76)	4.58 (3.24, 6.31)
Node 2	3.15 (2.31, 4.05)	3.14 (2.31, 4.02)	3.57 (2.33, 4.81)	3.58 (2.39, 4.85)	3.40 (1.93, 5.00)
Node 3	1.13 (0.78, 1.51)	1.16 (0.79, 1.53)	1.29 (0.81, 1.82)	1.37 (0.86, 1.94)	-
Node 4	1.38 (0.91, 1.86)	1.41 (0.96, 1.92)	1.55 (0.88, 2.34)	1.62 (0.92, 2.41)	-
Node 5	3.25 (2.46, 4.09)	3.24 (2.44, 4.05)	3.57 (2.36, 4.83)	3.55 (2.40, 4.73)	2.71 (1.39, 4.37)
Node 6	2.31 (1.74, 2.96)	2.32 (1.73, 2.94)	2.15 (1.41, 2.91)	2.20 (1.47, 2.95)	1.89 (1.01, 2.80)
Node 7	1.91 (1.41, 2.44)	1.93 (1.44, 2.46)	1.73 (1.20, 2.35)	1.80 (1.24, 2.42)	1.24 (0.67, 2.02)
Node 8	1.69 (1.23, 2.19)	1.71 (1.25, 2.20)	1.19 (0.79, 1.61)	1.27 (0.86, 1.72)	-

See Fig. 2 for respective node numbers; node 3 provides estimated ages of the focal Lake Ohrid group (UCLN-Y and *UCLN-BD (*BEAST species tree analysis) represent the favoured models, respectively; divergence times refer to mean, lower and upper 95% HPD values). Note that not all nodes are available in the *BEAST analyses

### Phylogeographic patterns

**Fig. 3:**
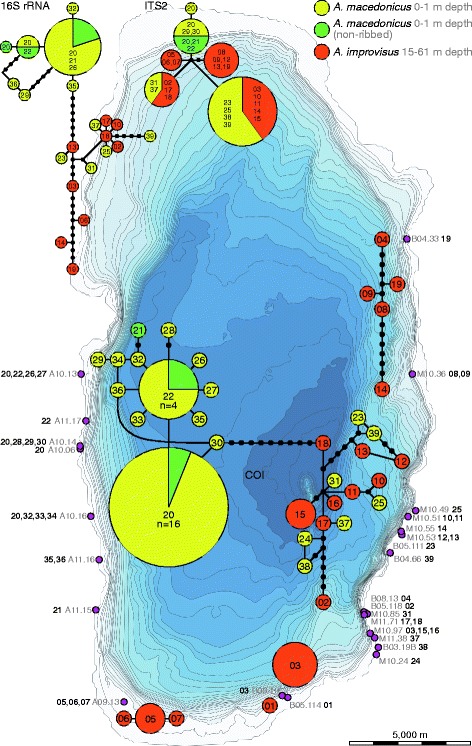
Parsimony networks for COI (*central*), 16S rRNA and ITS2 (*top*). The central COI haplotype network consists of one major and four minor networks. Position of haplotypes and haplotype groups for the COI dataset approximately refer to sampling sites across the lake. Numbers in circles refer to haplotype numbers shown in Additional file 1: Table S1. Sampling sites (pink) with locality numbers (grey) and haplotype numbers (black, bold), corresponding to the haplotype numbers shown in the networks

Three different parsimony network analyses were performed corresponding to the COI, 16S rRNA and ITS2 datasets (a reduced mitochondrial parsimony network based on 26 individuals is shown in Additional file 2: Figure S3). The haplotype network analysis revealed a highly diverse pattern for the COI dataset with a total number of 39 haplotypes in 5 networks (1 major: total number of haplotypes = 29, 4 minor: total number of haplotypes = 10) found among the two species and across the lake. For the 16S rRNA and the ITS2 dataset, only a reduced number of specimens was sequenced, which collapsed in 21 and 6 haplotypes, respectively. In general, the latter two networks plus the combined mitochondrial network revealed similar inter-specific and geographic patterns, though the resolution among and within subgroups, as identified in the COI dataset, was less pronounced, particularly for the nuclear ITS2 dataset (Fig. 3; see haplotype numbers in Additional file 1: Table S1).

Two important findings can be derived from the network analyses: First, the major COI haplotype network comprises both morphologically and ecologically different endemic species, the littoral *A. macedonicus* (including non-ribbed specimens that are only found along the Albanian shore; see morphological characteristics and taxonomic remarks in Additional file 2: Figures S4-S6) and the sublittoral *A. improvisus*. Further networks can be identified in the COI dataset for the deep-dwelling *A. improvisus* in the north-eastern and southern part of the lake (Fig. 3). These four minor haplotypes are not connected with the major network based on the 95% connection limit referring to a minimum of 13 mutational steps (data not shown): haplotype 1 and 3 (13 steps), haplotype 3 and 24 (13), haplotype 4 and 24 (13), haplotype 14 and 34 (13), and haplotype 5 and 34 (14; see Fig. 3 and Additional file 1: Table S1 for haplotype numbers). Secondly, the major COI network consists of two subgroups, one from the Albanian shore (western shoreline; *A. macedonicus*) and one comprising populations of both species occurring in a comparatively small geographic range along the south-eastern part of the lake (hereafter called ‘mixed group’; Fig. 3). The 16 unique haplotypes found in that ‘mixed group’ are not shared between the two species, however, they are partly separated only by very few mutational steps.

These two subgroups (Albanian/western vs. Macedonian/eastern shore) are genetically and spatially distinct. Haplotypes found along the western shoreline are absent along the eastern shoreline and vice versa. While particular haplotypes along the eastern shoreline are mostly site-specific, some of the haplotypes identified along the western shoreline can be found along stretches of c. 6 km (see Fig. 3; haplotype 20 found between sampling sites A10.13 and A10.16). Moreover, the maximum genetic distance within both subgroups differ considerably (west: 0.9%, east: 3.1%), even when *A. improvisus* is excluded from the ‘mixed group’ (2.0%).

## Discussion

### Divergence times of acroloxids and Europe’s palaeogeography since the Pliocene

Estimation of divergence times and topological patterns suggest that a widespread ancestral population of *Acroloxus* sp. originated in the Middle Pliocene and has existed in an area that included today’s northern Italy, the western Balkans and possibly even Anatolia (Fig. 2). After the closure of the Paratethys during the Pliocene, a land bridge was formed connecting Anatolia with the Balkans and giving rise to a continuous landmass, interrupted by several mountain chains such as the Dinarides-Hellenids, Rhodopes, Balkans, Carpathians, and the Alps [61–63]. The presence of suitable freshwater habitats in the Pliocene Euro-Mediterranean subregion is supported by both palaeogeographic reconstructions and a species-rich gastropod fossil record (e.g., [63–65]). Freshwater limpets of the family Acroloxidae are generally assumed to represent an ancient group, which may have originated in the Cretaceous or late Paleocene (e.g., [40, 66, 67]). While several fossil species have been assigned to the potentially older and morphologically different genus *Pseudancylastrum* (see [68]), central European and Anatolian fossils attributed to the genus *Acroloxus* are considerably younger (mainly from the Pliocene; e.g., [40, 69, 70]). Issues related to species assignment and potential sampling artefacts thus hamper the correlation between present distribution patterns and general palaeogeographic units identified for the gastropod fauna since the Miocene [63–65]. However, estimated divergence times for the MRCA of *Acroloxus* appear to be generally plausible in the light of palaeogeographic reconstructions and the first appearance of fossils in Europe.

### Biogeographic patterns and the origin of Lake Ohrid’s endemics

In view of the present phylogenetic relationships, reconstructing the biogeographic history of Euro-Mediterranean *Acroloxus* species remains challenging. Very surprisingly, the analyses identified *A. lacustris* populations from Lake Mergozzo (and the Ohrid endemics) to be the potential sister to the remaining *Acroloxus* species and populations examined, depending on the analysis performed (Fig. 2). Lake Mergozzo is part of the Lake Maggiore watershed, which is located in an area that has probably served as an interglacial refugium for cold-adapted species in the latest Pleistocene [71]. The geological origin of Lake Mergozzo is questionable as it may have either formed by Pleistocene glaciations or by Pliocene fluvial processes after the Messinian regression (e.g., [72]). However, given the estimated timeframe for the split between the Mergozzo and Ohrid clades (mean age: c. 3.58 My) and the first diversification within the Mergozzo clade (mean age: c. 1.62 My), the isolated position of Lake Mergozzo from the remaining (and slightly younger) central and western European *A. lacustris* populations is thus certainly not related to Quaternary processes. Our data may further suggest a northward colonization route out of Anatolia (including *A. egirdirensis* and *Acroloxus* sp. from Lake Kırkgöz) into central Europe (Fig. 2). However, testing the role of Lake Mergozzo as the source for the potential ancestral population for Lake Ohrid and the remaining Euro-Mediterranean species, and a northward colonization hypothesis out of Anatolia would require a denser sampling, particularly in central and western Europe.

Two major findings for the Lake Ohrid endemics emerge based on the phylogenetic relationships reconstructed. First, a non-Balkan origin has to be assumed for the acroloxid limpets given that neither the cave-dwelling *A. tetensi* from Slovenia nor the remaining populations from the Balkans (see Fig. 1), which all cluster within the very distinct *A. lacustris* clade, are closely related to the Lake Ohrid species (Fig. 2). This is in contrast to other biogeographic studies, suggesting that the endemic faunas of Lake Ohrid often show zoogeographic affinities to the western Balkans [38, 73–80]. Secondly, the monophyly of the endemic Ohrid *Acroloxus* species suggests a single colonization of Lake Ohrid that has occurred no later than c. 1.37 (0.86, 1.94) My ago. This roughly coincides with the estimated age of reaching deep-water conditions in the lake [12, 14], rendering the process of intra-lacustrine speciation most likely.

### Geography and ecology as key drivers for the diversification in Lake Ohrid

Speciation has been most often considered from a spatial perspective by assuming that species either evolve in allopatry, parapatry or sympatry in the presence or absence of physical barriers that can either restrict or allow gene flow between two populations to a particular extent (e.g., [81–83]). However, reproductive barriers can also be independent of geography (physical barriers) and may be fostered by ecologically-based divergent selection in different environments (reviewed in e.g., [82, 84–86]). Factors promoting a selection are manifold [82, 84] and may eventually lead to a complete reproductive isolation (particularly when several ecological dimensions are involved) along steep ecological or geographic clines [83]. Geographic barriers and ecological clines may be present in various freshwater water bodies, however, they are potentially more pronounced in large and deep environments with a variety of habitats and that experienced a long environmental history.

While sympatric speciation has been rarely tested in Lake Ohrid (but see [87]), the existence of physical barriers potentially giving rise to allopatric and parapatric speciation has been discussed before for Lake Ohrid [88]. Consequently, speciation involving a (micro-)geographic component has been attributed to the mode of parapatric speciation (see discussions in [13] and [86]). Parapatric speciation may occur i) along ecological gradients, ii) along geographic gradients (on a horizontal or vertical level) or iii) based on the mosaic distribution of suitable habitats (sensu [86]).

For Lake Ohrid, such gradients (i.e., horizontal and vertical zonations) have been already proposed by Hadžišče and Radoman [37, 76] and were reviewed in detail by Albrecht and Wilke [13]. Accordingly, the lake can be subdivided into five different horizontal zones that differ from each other by their geology, substrate and vegetation types, and the occurrence of sublacustrine springs [13, 89]. The combination of these different abiotic factors may explain why the eastern part shows a generally high habitat and mollusc diversity, while the western part is characterized by less diverse habitats and a depauperate mollusc fauna [90]. Interestingly, such a horizontal zonation only applies to the upper water column and could not be observed in deeper layers [17].

The horizontal distribution of acroloxids across the lake revealed that only the littoral *A. macedonicus* occurs along both the western and eastern shore, while the sublittoral *A. improvisus* could only be found in the north-eastern, eastern and southern parts of the lake (Fig. 3). The genetic data further revealed that the highest haplotype diversity is found in the south-eastern part (Fig. 3). This is particularly interesting because the area off Veli Dab (localities including haplotype numbers 10–14, 23, 25, and 39 in Fig. 3) represents one of the three biodiversity hotspots for gastropods in terms of both species richness and endemicity (see [17, 90]). However, the horizontal zonation proposed appears to have only a small impact on the distribution of both limpet species across the lake. One remarkable exception is the lack of *A. macedonicus* in shallower sandy stretches in the littoral of the northern and southern part. We here assume that these long stretches of soft substrate (see [89] and Fig. 1) represent unsuitable habitats for hard substrate-dwelling species. Therefore, these areas may not only be responsible for the comparatively low mollusc species richness in the littoral in general (see [90]), but may have also impeded gene flow between the two geographically isolated *A. macedonicus* groups (western vs. eastern).

Albrecht and Wilke [13] further suggest a vertical zonation within Lake Ohrid based on previous observations by Radoman [76] and including five zones differing in temperature range, sunlight penetration, substratum, vegetation, and water movement. However, other studies suggest a less complex vertical zonation based on both physical attributes and gastropod species distribution [90], and showed that some of horizontal and vertical zones considerably overlap [17]. Nonetheless, two potential physical barriers, namely the ‘*Chara* belt’ and the ‘shell zone’ are of particular importance for the present study.

The ‘*Chara* belt’ has been assumed to represent a moderate to strong physical barrier for particular species by forming a dense net with potentially anoxic or even toxic interstitial water [13, 38]. Although this belt is only heterogeneously distributed across the lake (e.g., [91]), such a physical barrier could prevent dispersal from the upper littoral to the lower sublittoral and thus may have triggered (micro-)geographic speciation as already suggested by Hubendick and Radoman [36, 76]. The same might apply to the so-called ‘shell zone’, a bed of *Dreissena* shells in 20–35 m water depth that potentially forms a physical barrier for some invertebrate groups [13]. The existence of such physical barriers is mainly suggested based on the occurrence of putative species pairs of pulmonate and hydrobiid snails inhabiting different bathymetric layers [36, 38, 76, 88]. Unfortunately, only few sample areas exist in Lake Ohrid that enable testing for a vertical (bathymetric) separation in these two species. The south-eastern part represents an area where both species (partly) co-occur and show their highest abundance and genetic diversity (Fig. 3). However, the mitochondrial-based haplotype networks exhibit only very few mutational steps between the species-specific haplotypes and revealed a ‘mixed group’ comprising both species.

Based on the above-mentioned observations, we conclude that both geography and ecology have played a major role for the distribution and diversification in Lake Ohrid’s morphologically and ecologically distinct freshwater limpets and suggest the following scenarios. Geography is certainly important, particularly on a horizontal dimension, resulting in independent genetic lineages/clades in both species found along different shorelines and that are potentially geographically separated by unsuitable habitats. On a vertical level, the patterns found suggest that geographic separation has been the main evolutionary process as suggested for other taxonomic groups in Lake Ohrid (see [13, 88]) and for limpets endemic to Lake Baikal [35]. However, genetic differentiation can be weak between and within the two Ohrid species when the geographic distance is low. This is particularly the case for the south-eastern part, where the slope is considerably steeper compared to other areas in the lake such as the northern and southern shorelines (Fig. 1). This geographic/bathymetric characteristic plus the weakness of the above-mentioned physical barriers may explain why the genetic analyses revealed a ‘mixed clade’, comprising several haplotypes of both species, and in which the variation among species is not higher than within species (Fig. 3). These data suggest that none of the physical barriers proposed (‘*Chara* belt’ and ‘shell zone’) have completely prevented gene flow between *Acroloxus* species endemic to Lake Ohrid and/or that the low genetic differentiation observed is related to an early phase of speciation and thus involves ancestral polymorphism.

From a different evolutionary perspective, the present pattern could also indicate a case of ecological speciation due to divergent natural selection in different environments [84]. Findings supporting this hypothesis include the presence of morphologically distinct species (predominantly sculptured shells in the littoral vs. smooth shells in the sublittoral) that are adapted to different environments, the moderate to strong genetic differentiation between the two species, and a steep ecological cline across the water column (littoral–sublittoral). According to Nosil et al. [84], such steep ecological clines may indicate that the process of speciation may have completed. In fact, the onset of intralacustrine diversification-leading to distinct littoral and sub-littoral forms-roughly coincided with the establishment of deep-water conditions in Lake Ohrid c. 1.3 Mya.

Integrating the morphological, ecological and molecular evidences, we find it plausible to argue that an initial differentiation of shallow and deep-water forms (ecological speciation) was subsequently overlaid by geographic processes driven by physical barriers and restricted habitat availability.

## Conclusions

This study provides a first molecular phylogeny for freshwater limpets of the genus *Acroloxus* inhabiting the Euro-Mediterranean subregion, with particular focus on the Balkan Lake Ohrid-the oldest freshwater ancient lake in Europe and a hotspot of biodiversity. Freshwater limpets of the genus *Acroloxus* have presumably colonized Lake Ohrid once and started to diversify when the lake reached deep-water conditions. Interestingly, the endemic Ohrid species are not closely related to the common, widespread and slightly younger European *A. lacustris*, but rather represent a distinct reciprocally monophyletic group that may be closely related to populations found in the Italian Lake Mergozzo. Moreover, these phylogenetic relationships suggest that the Balkan region has probably not served as the ancestral area, contrary to other endemic freshwater groups.

Based on the strong morphological and ecological differences and genetic patterns observed, we conclude that two endemic species occur in Lake Ohrid, namely the littoral *A. macedonicus* and the sublittoral *A. improvisus*. Moreover, we hypothesize that possibly both ecological (along a vertical habitat gradient) and geographic (spatial isolation on a horizontal scale, patchiness of suitable habitats, and low mobility of the populations) speciation gave rise to the two different species, though a clear distinction between these two modes poses a significant challenge. However, assuming that the different shell morphology and ecology are conservative features, it seems reasonable to assume that ecological speciation along a vertical habitat gradient may have been the predominant process in the early stage of speciation, triggered by the onset of deep-water conditions. Subsequent geographic processes then gave rise to the phylogeographic patterns observed today. The weak genetic differentiation found between these two species also suggests an early stage of speciation (incipient speciation), irrespective of which mode (ecological or geographic) is predominating, and thus provides a fascinating model system for testing ongoing diversification and speciation processes in Lake Ohrid using high-resolution genetic markers in future studies.

## Additional files

Additional file 1: Table S1. Specimens examined including locality information and GenBank accession numbers. (PDF 166 kb)
Additional file 2: Figure S1. MCC trees for the four BEAST analyses performed (outgroup removed). See Methods and Results for details. Figure S2 MCC trees for the four *BEAST analyses performed (including outgroups). See Methods and Results for details. **Figure S3.** Combined mitochondrial parsimony network for the two markers 16S rRNA and COI. Position of haplotypes and haplotype groups approximately refer to sampling sites across the lake (compare Fig. 3; yellow: *Acroloxus macedonicus*; green: non-ribbed *A. macedonicus*; orange: *A. improvisus*). Regular numbers refer to DNA voucher numbers; COI haplotype numbers are marked with a hashtag (see Additional file 1: Table S1). **Figure S4.** The shell of the regular (ribbed) *Acroloxus macedonicus* (SEM data). A–G, J–K — protoconch (C, F–G — initial plate; E, J–K — sculpture). H–I, L–M — teleoconch (L–M — fragments of ribbed surface). A–B, E, G–H — left view; C–D, I — top view; F — posterior-right view. Scale bars: A– D, F–G, J, L–M = 0.1 mm, E = 0.05 mm, H–I = 1 mm, K = 10 μm. **Figure S5.** The shell of non-ribbed specimens of *Acroloxus macedonicus* (SEM data). 1 — first specimen, 2 — second spm. 1A–C, 1E, 2A–C — teleoconch (1E — fragment of smooth surface). 1D, 2D — protoconch. 1A, 2A — left view; 1B, 2B — rear view; 1C–D, 2C–D — top view. Scale bars: 1A– D, 2A–D = 1 mm, 1E = 0.1 mm. **Figure S6.** The shell of *Acroloxus improvisus* (SEM data). A–F, I–L — protoconch (E, J, K–L — sculpture; F, I — initial plate). G–H, M — teleoconch (M — fragment of smooth surface). A — posterior-left view; B, F–G — left view; C — right view; D, H — top view; I — right and top view. Scale bars: A–F, I–K, M = 0.1 mm, G–H = 1 mm, L = 10 μm. (PDF 39654 kb)

## Abbreviations

16S rRNA: 16S ribosomal RNA
26S rRNA: 16S ribosomal RNA
AIC: Akaike Information Criterion
AICc: Corrected Akaike Information Criterion
BF: Bayes factor
COI: Cytochrome c oxidase subunit I
H3: Histone 3
HPD: Highest posterior density
ITS2: Internal transcribed spacer 2
MCC: Maximum clade credibility
My: Million years
Mya: Million years ago
PCR: Polymerase chain reaction
STR-BD: Strict clock, birth-death process
STR-Y: Strict clock, Yule process
UCLN-BD: Uncorrelated lognormal relaxed clock, birth-death process
UCLN-Y: Uncorrelated lognormal relaxed clock, Yule process
