# Supplementary material for: Origin and diversification of Lake Ohrid’s endemic acroloxid limpets: the role of geography and ecology
Source: BMC Evol Biol. 2016 Dec 15;16:273. doi: 10.1186/s12862-016-0826-6 (PMC5159953; doi:10.1186/s12862-016-0826-6)
Supplement: Additional file 1: Table S1. — Specimens examined including locality information and GenBank accession numbers. (PDF 166 kb) [file 12862_2016_826_MOESM1_ESM.pdf]

Table S1. Specimens examined including locality information and GenBank accession numbers.

Taxa	Locality	Coordinates (latitude, longitude)	Locality no.	DNA voucher (UGSB no.)	16S acc. no.	28S acc. no.	COI acc. no. haplo. no.	H3 acc. no.	ITS2 acc. no.
**Outgroup**									
Latia neritoides	Blue Bull stream, North Island, New Zealand; leg. and det. M. Haase; 21 Apr 2003	-37.923300, 175.088300		F207 (17668)	EF489307	FJ917245	EF489384	KY092831	–
Lymnaea stagnalis	River Gera near mouth, Gebesee, Thuringia, Germany; leg. and det. C. Albrecht; 27 Oct 2001	51.126090, 10.930460		F22 (17669)	EF489314	EF489367	EF489390	KY092832	–
Planorbarius corneus	dead river of River Alte Oder, Altranft, Brandenburg, Germany; leg. and det. C. Albrecht; date 02 Nov 2001	52.766820, 14.099370		F27 (17670)	JN794123	KY092721	AY282590	KY092833	–
Acroloxus									–
A. egirdirensis	Lake Eğirdir, Isparta, Turkey; stones; leg. M.Z. Yıldırım, Ü. Kebapçi, S. Koşal Şahin, det. A.A. Shirokaya; 17 Mar 2005	37.892000, 30.902000	RM05.91	4332 (9529)	KY092673	KY092722	JQ646077	KY092834	–
	Lake Eğirdir, Isparta, Turkey; stones; leg. M.Z. Yıldırım, Ü. Kebapçi, S. Koşal Şahin, det. A.A. Shirokaya; 17 Mar 2005	37.892000, 30.902000	RM05.91	4333 (9530)	KY092674	KY092723	JQ646078	KY092835	–
A. improvisus	Lake Ohrid, Sv. Naum, Macedonia; 15-25 m depth; leg. C. Albrecht, T. Geertz, A. Hauswald; det. C. Albrecht; 03 Oct 2005	40.948830, 20.773910	B05.114	4870 (9871)	–	KY092724	KY092753 01	KY092836	–
	Lake Ohrid, Sv. Naum, Macedonia; 15-25 m depth; leg. C. Albrecht, T. Geertz, A. Hauswald; det. C. Albrecht; 03 Oct 2005	40.948830, 20.773910	B05.118	4957 (9873)	KY092675	–	KY092754 02	–	KY092865
	Lake Ohrid, Sv. Naum, Macedonia; 9.4-30 m depth; leg. and det. C. Wolff, P. von Oheimb, K. Heiler, S. Nachtigall, D. Lohfink; 21 May 2008	40.918160, 20.732980	B08.19	10177 (17671)	KY092676	–	KY092755 03	–	KY092866
	Lake Ohrid, Sv. Naum, Macedonia; 9.4-30 m depth; leg. and det. C. Wolff, P. von Oheimb, K. Heiler, S. Nachtigall, D. Lohfink; 21 May 2008	40.918160, 20.732980	B08.13	10180 (9881)	–	KY092725	KY092756 04	KY092837	–
	Lake Ohrid, Pogradec, Albania; 5-25 m depth; leg. K. Preußer, M. Richter, N. Tron; det. C. Albrecht; 28 May 2009	40.915790, 20.655330	A09.13	11780 (9883)	–	–	KY092757 05	–	KY092867
	Lake Ohrid, Pogradec, Albania; 5-25 m depth; leg. K. Preußer, M. Richter, N. Tron; det. C. Albrecht; 28 May 2009	40.915790, 20.655330	A09.13	11781 (17672)	–	–	KY092758 05	–	KY092868
	Lake Ohrid, Pogradec, Albania; 5-25 m depth; leg. K. Preußer, M. Richter, N. Tron; det. C. Albrecht; 28 May 2009	40.915790, 20.655330	A09.13	11782 (9884)	KY092677	KY092726	KY092759 06	KY092838	KY092869
	Lake Ohrid, Pogradec, Albania; 5-25 m depth; leg. K. Preußer, M. Richter, N. Tron; det. C. Albrecht; 28 May 2009	40.915790, 20.655330	A09.13	11783 (9885)	–	–	KY092760 07	–	–
	Lake Ohrid, Eleshec, Macedonia; 40-48 m depth; leg. C. Clewing, K. Schreiber, J. Lorenschat; det. C. Albrecht; 21 Feb 2012	41.037190, 20.796960	M10.36	13485 (9888)	–	–	KY092761 08	–	KY092870
	Lake Ohrid, Eleshec, Macedonia; 40-48 m depth; leg. C. Clewing, K. Schreiber, J. Lorenschat; det. C. Albrecht; 21 Feb 2012	41.037190, 20.796960	M10.36	13486 (9889)	–	–	KY092762 09	–	KY092871
	Lake Ohrid, Gradishte, Macedonia; 40-60 m depth; leg. T. Hauffe, C. Wolff; det. C. Albrecht; 23 Feb 2012	40.984440, 20.794830	M10.51	13488 (9890)	KY092678	–	KY092763 10	–	KY092872
	Lake Ohrid, Gradishte, Macedonia; 40-60 m depth; leg. T. Hauffe, C. Wolff; det. C. Albrecht; 23 Feb 2012	40.984440, 20.794830	M10.51	13489 (9891)	–	–	KY092764 11	–	KY092873
	Lake Ohrid, north of Veli Dab, Macedonia; 25 m depth; leg. T. Hauffe, C. Wolff; det. C. Albrecht; 23 Feb 2012	40.977710, 20.791810	M10.53	13493 (9892)	–	–	KY092765 12	–	KY092874
	Lake Ohrid, north of Veli Dab, Macedonia; 25 m depth; leg. T. Hauffe, C. Wolff; det. C. Albrecht; 23 Feb 2012	40.977710, 20.791810	M10.53	13494 (9893)	KY092679	–	KY092766 13	–	KY092875
	Lake Ohrid, north of Veli Dab, Macedonia; 15 m depth; leg. T. Hauffe, C. Wolff; det. C. Albrecht; 23 Feb 2012	40.978980, 20.791430	M10.55	13496 (9894)	KY092680	–	KY092767 14	–	KY092876
	Lake Ohrid, southeast end of lake, Macedonia; 20-26 m depth; leg. A.A. Shirokaya, A. Temmen, D. Delicado; det. A.A. Shirokaya; 07 Sep 2010	40.941250, 20.776210	M10.97	14540 (9902)	–	–	KY092768 15	–	KY092877
	Lake Ohrid, southeast end of lake, Macedonia; 20-26 m depth; leg. A.A. Shirokaya, A. Temmen, D. Delicado; det. A.A. Shirokaya; 07 Sep 2010	40.941250, 20.776210	M10.97	14541 (17673)	–	–	KY092769 15	–	KY092878
	Lake Ohrid, southeast end of lake, Macedonia; 20-26 m depth; leg. A.A. Shirokaya, A. Temmen, D. Delicado; det. A.A. Shirokaya; 07 Sep 2010	40.941250, 20.776210	M10.97	14542 (9903)	–	–	KY092770 16	–	–
	Lake Ohrid, southeast end of lake, Macedonia; 20-26 m depth; leg. A.A. Shirokaya, A. Temmen, D. Delicado; det. A.A. Shirokaya; 07 Sep 2010	40.941250, 20.776210	M10.97	14543 (17674)	–	–	KY092771 03	–	–
	Lake Ohrid, southeast end of lake, Macedonia; 20-26 m depth; leg. A.A. Shirokaya, A. Temmen, D. Delicado; det. A.A. Shirokaya; 07 Sep 2010	40.941250, 20.776210	M10.97	14544 (9904)	–	–	KY092772 03	–	–

Taxa	Locality	Coordinates (latitude, longitude)	Locality no.	DNA voucher (UGSB no.)	16S acc. no.	28S acc. no.	COI acc. no. haplo. no.	H3 acc. no.	ITS2 acc. no.
	Lake Ohrid, Sv. Naum, Macedonia; leg. T. Hauffe, C. Wolff; det. T. Hauffe; 11 May 2011	40.948090, 20.773300	M11.71	17239 (17675)	KY092681	–	KY092773<br>17	–	KY092879
	Lake Ohrid, Sv. Naum, Macedonia; leg. T. Hauffe, C. Wolff; det. T. Hauffe; 11 May 2011	40.948090, 20.773300	M11.71	17240 (17676)	KY092682	–	KY092774<br>18	–	KY092880
	Lake Ohrid, Gorica hill, Macedonia; 15-30 m depth; leg. T. Wilke, M. Gerhardt, D. Lohfink; det. T. Hauffe; 18 Sep 2004	41.087140, 20.794240	B04.33	F393 (9863)	KY092683	KY092727	KY092775<br>19	KY092839	KY092881
A. lacustris	Lake Prespa, bay on cliff-like coast, Greece; 0-60 cm depth, rocks; leg. and det. T. Wilke, C. Albrecht; 16 May 2005	40.820320, 21.019390	B05.45	4521 (9867)	KY092687	KY092730	KY092779	KY092842	–
	Lake Mikri Prespa, Mikrolimni, Greece; 0-30 cm depth, rocks; leg. and det. T. Wilke, C. Albrecht; 15 May 2005	40.732140, 21.104740	B05.39	4525 (9868)	KY092688	KY092731	KY092780	KY092843	–
	Lake Vegoritida, peninsula opposite Amissa, Greece; 0-2 m depth, rocks; leg. and det. T. Wilke, C. Albrecht; 10 May 2005	40.786190, 21.816850	B05.07	4533 (9869)	KY092689	KY092732	KY092781	KY092844	–
	Lake Prespa, c. 1 km north of Psarades, Greece; 10 m depth, rocks, mud; leg. and det. T. Wilke, C. Albrecht; 16 May 2005	40.836770, 21.021410	B05.46	4534 (9870)	KY092690	KY092733	KY092782	KY092845	–
	Valjevac, Zasavica, Serbia; leg. and det. P. Glöer; 09 Jun 2006	44.942842, 19.513075*	RM07.13	6645 (17249)	KY092691	KY092734	KY092783	KY092846	–
	Lake Mergozzo, east shore, Mergozzo, Italy; 20 cm depth, rocks, stone, detritus; leg. and det. U. Bößneck, K. Bößneck, T. Geertz, R. Schultheiß, T. Hauffe; 05 Apr 2008	45.955300, 8.475230	IT08.01	9399 (17678)	KY092692	KY092735	KY092784	KY092847	–
	Lake Mergozzo, east shore, Mergozzo, Italy; 20 cm depth, rocks, stone, detritus; leg. and det. U. Bößneck, K. Bößneck, T. Geertz, R. Schultheiß, T. Hauffe; 05 Apr 2008	45.955300, 8.475230	IT08.01	9400 (17679)	KY092693	KY092736	KY092785	KY092848	–
	Lake Ohrid, Ohrid, Macedonia; 10-50 cm depth, rocks, stones, sand, sapropel; leg. and det. A.A. Shirokaya, A. Temmen, D. Delicado, C. Marek, K. Preußer, T. Hauffe, K. Schreiber; 12 Sep 2010	41.116188, 20.788065	M10.128	14520 (17680)	KY092694	KY092737	KY092786	KY092849	–
	Lake Mergozzo, Mergozzo, Italy; 0-5 m depth; leg. and det. C. Albrecht; 23 Mar 2009	45.959420, 8.468300	ITA2009-2	22706 (17238)	KY092695	KY092738	KY092787	KY092850	–
	Lake Mergozzo, Mergozzo, Italy; 0-5 m depth; leg. and det. C. Albrecht; 23 Mar 2009	45.959420, 8.468300	ITA2009-2	22707 (17239)	KY092696	KY092739	KY092788	KY092851	–
	Lake Mergozzo, Mergozzo, Italy; 0-5 m depth; leg. and det. C. Albrecht; 23 Mar 2009	45.960380, 8.467130	ITA2009-5	22708 (17241)	KY092697	KY092740	KY092789	KY092852	–
	Gülper Havel at bridge between the village of Gülpe and the ecological station, Gülpe, Havelland, Brandenburg, Germany; leg. and det. C. Albrecht, T. Wilke; 31 Jul 2005	52.726390, 12.218300	CA2005.29	22710 (17245)	KY092698	KY092741	KY092790	KY092853	–
	Artificial lake at Šum spring, Macedonia; leg. and det. Z. Fehér	41.182833, 20.632333	94.802	22712 (17248)	KY092699	KY092742	KY092791	KY092854	–
	Lake Schloßsee, Harnekop, Brandenburg, Germany; 10 cm depth, sand, mud; leg. and det. C. Albrecht; 02 Nov 2001	52.687003, 14.006000	CA33.01	F19 (16896)	EF489311	EF489364	AY282581	KR822570	–
	Eastern bank of artificial lake at Šum spring, Macedonia; stones, mud; leg. and det. T. Wilke, C. Albrecht, R. Schultheiß, M. Gerhardt, D. Lohfink; 19 Sep 2004	41.184050, 20.633500	B04.51	F348 (17681)	KY092700	KY092743	KY092792	KY092855	–
	Lake Ohrid, Tusemište (spring complex), Qark Korça, Albania; 50 cm depth, mud; leg. and det. C. Albrecht and R. Schultheiß; 18 Sep 2004	40.898600, 20.712397	B04.43	F365 (9862)	KR822493	KR822514	KR822541	KR822571	–
	Lake Summerville Lough, County Galway, Ireland; leg. D. Ó Foighil	53.498333, -8.601667	-		DQ103751[#]	DQ328296[#]	DQ328271[#]		–
A. macedonicus (non-ribbed)	Lake Ohrid, western shore, Albania; 0-50 cm depth, rocks; leg. and det. C. Clewing, K. Schreiber, J. Lorenschat, C. Wolff, T. Hauffe; 20 Feb 2010	41.009520, 20.633820	A 10.06	12989 (17677)	KY092684	KY092728	KY092776<br>20	KY092840	KY092882
	Lake Ohrid, western shore, Albania; leg. K. Schreiber, C. Clewing, J. Föller; det. T. Hauffe; 11 May 2011	40.949900, 20.641480	A11.15	17232 (8270)	KY092685	–	KY092777<br>21	–	KY092883
	Lake Ohrid, western shore, near Hotel/Restaurant “Lean”, Albania; leg. K. Schreiber, C. Clewing, J. Föller; det. T. Hauffe; 11 May 2011	41.019860, 20.636610	A11.17	17233 (8178)	KY092686	KY092729	KY092778<br>22	KY092841	KY092884
A. macedonicus	Lake Ohrid, Veli Dab, Macedonia; leg. C. Albrecht, T. Geertz, A. Hauswald; det. C. Albrecht; 02 Oct 2005	40.971060, 20.786030	B05.111	4956 (9872)	KY092701	KY092744	KY092793<br>23	KY092856	KY092885
	Lake Ohrid, southeast end of lake, Macedonia; leg. C. Clewing, K. Schreiber; det. C. Albrecht; 20 Feb 2012	40.933350, 20.779250	M10.24	12987 (9886)	–	–	KY092794<br>24	–	–
	Lake Ohrid, western shore, Albania; 0-50 cm depth, rocks; leg. and det. C. Clewing, K. Schreiber, J. Lorenschat, C. Wolff, T. Hauffe; 20 Feb 2010	41.009520, 20.633820	A10.06	12988 (17682)	–	–	KY092795<br>20	–	KY092886
	Lake Ohrid, Gradishte, Macedonia; leg. T. Hauffe, C. Wolff; det. C. Albrecht; 23 Feb 2012	40.986640, 20.798570	M10.49	12995 (9887)	KY092702	–	KY092796<br>25	–	KY092887
	Lake Ohrid, south of Lin, Albania; leg. D. Delicado, A.A. Shirokaya, K. Schreiber, K. Preußer; det. A.A. Shirokaya; 09 Sep 2010	41.037190, 20.634260	A10.13	14521 (17683)	–	–	KY092797<br>22	–	–

Taxa	Locality	Coordinates (latitude, longitude)	Locality no.	DNA voucher (UGSB no.)	16S acc. no.	28S acc. no.	COI acc. no. haplo. no.	H3 acc. no.	ITS2 acc. no.
	Lake Ohrid, south of Lin, Albania; leg. D. Delicado, A.A. Shirokaya, K. Schreiber, K. Preußer; det. A.A. Shirokaya; 09 Sep 2010	41.037190, 20.634260	A10.13	14522 (17684)	–	–	KY092798 22	–	–
	Lake Ohrid, south of Lin, Albania; leg. D. Delicado, A.A. Shirokaya, K. Schreiber, K. Preußer; det. A.A. Shirokaya; 09 Sep 2010	41.037190, 20.634260	A10.13	14523 (9895)	KY092703	–	KY092799 26	–	–
	Lake Ohrid, south of Lin, Albania; leg. D. Delicado, A.A. Shirokaya, K. Schreiber, K. Preußer; det. A.A. Shirokaya; 09 Sep 2010	41.037190, 20.634260	A10.13	14524 (17685)	–	–	KY092800 22	–	–
	Lake Ohrid, south of Lin, Albania; leg. D. Delicado, A.A. Shirokaya, K. Schreiber, K. Preußer; det. A.A. Shirokaya; 09 Sep 2010	41.037190, 20.634260	A10.13	14525 (17686)	–	–	KY092801 20	–	–
	Lake Ohrid, south of Lin, Albania; leg. D. Delicado, A.A. Shirokaya, K. Schreiber, K. Preußer; det. A.A. Shirokaya; 09 Sep 2010	41.037190, 20.634260	A10.13	14527 (9896)	–	–	KY092802 27	–	–
	Lake Ohrid, south of Lin, Albania; leg. D. Delicado, A.A. Shirokaya, K. Schreiber, K. Preußer; det. A.A. Shirokaya; 09 Sep 2010	41.010570, 20.633750	A10.14	14528 (9897)	–	–	KY092803 28	–	–
	Lake Ohrid, south of Lin, Albania; leg. D. Delicado, A.A. Shirokaya, K. Schreiber, K. Preußer; det. A.A. Shirokaya; 09 Sep 2010	41.010570, 20.633750	A10.14	14529 (17687)	–	–	KY092804 20	–	–
	Lake Ohrid, south of Lin, Albania; leg. D. Delicado, A.A. Shirokaya, K. Schreiber, K. Preußer; det. A.A. Shirokaya; 09 Sep 2010	41.010570, 20.633750	A10.14	14530 (17688)	–	–	KY092805 20	–	KY092888
	Lake Ohrid, south of Lin, Albania; leg. D. Delicado, A.A. Shirokaya, K. Schreiber, K. Preußer; det. A.A. Shirokaya; 09 Sep 2010	41.010570, 20.633750	A10.14	14531 (17689)	–	–	KY092806 20	–	–
	Lake Ohrid, south of Lin, Albania; leg. D. Delicado, A.A. Shirokaya, K. Schreiber, K. Preußer; det. A.A. Shirokaya; 09 Sep 2010	41.010570, 20.633750	A10.14	14532 (17690)	KY092704	KY092745	KY092807 20	KY092857	–
	Lake Ohrid, south of Lin, Albania; leg. D. Delicado, A.A. Shirokaya, K. Schreiber, K. Preußer; det. A.A. Shirokaya; 09 Sep 2010	41.010570, 20.633750	A10.14	14533 (9898)	KY092705	–	KY092808 29	–	KY092889
	Lake Ohrid, south of Lin, Albania; leg. D. Delicado, A.A. Shirokaya, K. Schreiber, K. Preußer; det. A.A. Shirokaya; 09 Sep 2010	41.010570, 20.633750	A10.14	14534 (9899)	–	–	KY092809 30	–	KY092890
	Lake Ohrid, south of Lin, Albania; leg. D. Delicado, A.A. Shirokaya, K. Schreiber, K. Preußer; det. A.A. Shirokaya; 09 Sep 2010	41.010570, 20.633750	A10.14	14535 (17691)	–	–	KY092810 20	–	–
	Lake Ohrid, south of Lin, Albania; leg. D. Delicado, A.A. Shirokaya, K. Schreiber, K. Preußer; det. A.A. Shirokaya; 09 Sep 2010	41.010570, 20.633750	A10.14	14536 (17692)	–	–	KY092811 20	–	–
	Lake Ohrid, south of Lin, Albania; leg. D. Delicado, A.A. Shirokaya, K. Schreiber, K. Preußer; det. A.A. Shirokaya; 09 Sep 2010	41.010570, 20.633750	A10.14	14537 (9900)	–	–	KY092812 20	–	–
	Lake Ohrid, Sv. Naum, Macedonia; leg. A.A. Shirokaya, A. Temmen, D. Delicado; det. A.A. Shirokaya; 06 Sep 2010	40.948390, 20.774790	M10.85	14539 (9901)	KY092706	–	KY092813 31	–	KY092891
	Lake Ohrid, western shore, near Restaurant "Kaltrina", Albania; leg. D. Delicado, A.A. Shirokaya, K. Schreiber; det. A.A. Shirokaya; 09 Sep 2010	40.984560, 20.639030	A10.16	14545 (17693)	–	–	KY092814 20	–	–
	Lake Ohrid, western shore, near Restaurant "Kaltrina", Albania; leg. D. Delicado, A.A. Shirokaya, K. Schreiber; det. A.A. Shirokaya; 09 Sep 2010	40.984560, 20.639030	A10.16	14546 (17694)	–	–	KY092815 20	–	–
	Lake Ohrid, western shore, near Restaurant "Kaltrina", Albania; leg. D. Delicado, A.A. Shirokaya, K. Schreiber; det. A.A. Shirokaya; 09 Sep 2010	40.984560, 20.639030	A10.16	14547 (17695)	KY092707	–	KY092816 20	–	–
	Lake Ohrid, western shore, near Restaurant "Kaltrina", Albania; leg. D. Delicado, A.A. Shirokaya, K. Schreiber; det. A.A. Shirokaya; 09 Sep 2010	40.984560, 20.639030	A10.16	14549 (17696)	KY092708	–	KY092817 20	–	–
	Lake Ohrid, western shore, near Restaurant "Kaltrina", Albania; leg. D. Delicado, A.A. Shirokaya, K. Schreiber; det. A.A. Shirokaya; 09 Sep 2010	40.984560, 20.639030	A10.16	14550 (9905)	KY092709	–	KY092818 32	–	–
	Lake Ohrid, western shore, near Restaurant "Kaltrina", Albania; leg. D. Delicado, A.A. Shirokaya, K. Schreiber; det. A.A. Shirokaya; 09 Sep 2010	40.984560, 20.639030	A10.16	14551 (9906)	–	–	KY092819 33	–	–
	Lake Ohrid, western shore, near Restaurant "Kaltrina", Albania; leg. D. Delicado, A.A. Shirokaya, K. Schreiber; det. A.A. Shirokaya; 09 Sep 2010	40.984560, 20.639030	A10.16	14552 (17697)	–	–	KY092820 20	–	–
	Lake Ohrid, western shore, near Restaurant "Kaltrina", Albania; leg. D. Delicado, A.A. Shirokaya, K. Schreiber; det. A.A. Shirokaya; 09 Sep 2010	40.984560, 20.639030	A10.16	14553 (17698)	KY092710	–	KY092821 20	–	–
	Lake Ohrid, western shore, near Restaurant "Kaltrina", Albania; leg. D. Delicado, A.A. Shirokaya, K. Schreiber; det. A.A. Shirokaya; 09 Sep 2010	40.984560, 20.639030	A10.16	14554 (9907)	–	–	KY092822 34	–	–
	Lake Ohrid, western shore, at camping site, Albania; leg. K. Schreiber, C. Clewing, J. Föller; det. T. Hauffe; 11 Apr 2011	40.968490, 20.643110	A11.16	17230 (17699)	KY092711	–	KY092823 35	–	–

Taxa	Locality	Coordinates (latitude, longitude)	Locality no.	DNA voucher (UGSB no.)	16S acc. no.	28S acc. no.	COI acc. no. haplo. no.	H3 acc. no.	ITS2 acc. no.
	Lake Ohrid, western shore, at camping site, Albania; leg. K. Schreiber, C. Clewing, J. Föller; det. T. Hauffe; 11 Apr 2011	40.968490, 20.643110	A11.16	17231 (17700)	KY092712	–	KY092824 36	–	–
	Lake Ohrid, sub-lacustrine springs, Macedonia; leg. K. Schreiber, C. Clewing, C. Wolff; det. T. Hauffe; 10 Apr 2011	40.939590, 20.778440	M11.38	17241 (7978)	KY092713	–	KY092825 37	–	KY092892
	Lake Ohrid, eastern shore, Macedonia; 2-3 m depth, rocks; leg. and det. C. Albrecht, T. Wilke; 19 May 2003	40.936020, 20.780230	B03.19B	F320 (9860)	–	–	KY092826 38	–	KY092893
	Lake Ohrid, bay near Veli Dab, Macedonia; 50 cm depth, stones; leg. and det. T. Wilke, C. Albrecht, R. Schultheiß, M. Gerhardt, D. Lohfink; 21 Sep 2004	40.970970, 20.786040	B04.66	F358 (9861)	KY092714	KY092746	KY092827 39	KY092858	KY092894
A. tetensi	Cave Planinska jama, Slovenia; leg. and det. B. Sket; 15 Oct 2009	45.820270, 14.245820	-	10080 (9879)	KY092715	KY092747	KY092828	KY092859	–
	Cave Planinska jama, Slovenia; leg. and det. B. Sket; 15 Oct 2009	45.820270, 14.245820	-	10081 (9880)	KY092716	KY092748	KY092829	KY092860	–
Acroloxus sp.	Lake Uluabat, between D200 and town Gölyazı, Karacabey, Bursa, Turkey; leg. and det. G. Hartz; 03 Apr 2008	40.185200, 28.689200	H50	9418 (2799)	KY092717	KY092749	KY092830	KY092861	–
	Lake Uluabat, 3.5 km east of the pumping station of the village Uluabat, Karacabey, Bursa, Turkey; leg. and det. G. Hartz; 02 Apr 2008	40.180600, 28.474000	H48	10018 (2928)	KY092718	KY092750	JQ646079	KY092862	–
	Lake Kırkgöz, at pumpstation, Döşemealtı, Turkey; leg. T. Wilke, T. Hauffe, D. Delicado; det. K. Schreiber; 24 Sep 2010	37.100570, 30.584710	TUR10.13	17235 (5897)	KY092719	KY092751	JQ646082	KY092863	–
	Lake Kırkgöz, Döşemealtı, Turkey; leg. T. Wilke, T. Hauffe, D. Delicado; det. K. Schreiber; 24 Sep 2010	37.109790, 30.580710	TUR10.09	17236 (8371)	KY092720	KY092752	JQ646083	KY092864	–

* coordinates estimated with GoogleEarth

# from [1,2]

1. Walther AC, Lee T, Burch JB, Ó Foighil D. *Acroloxus lacustris* is not an ancylid: a case of misidentification involving the cryptic invader *Ferrissia fragilis* (Mollusca: Pulmonata: Hygrophila). Mol Phylogenet Evol. 2006;39:271–5.

2. Walther AC, Lee T, Burch JB, Foighil DO. *E Pluribus Unum*: A phylogenetic and phylogeographic reassessment of *Laevapex* (Pulmonata: Ancylidae), a North American genus of freshwater limpets. Mol Phylogenet Evol. 2006;40:501–16.
