# Supplementary material for: Origin and diversification of Lake Ohrid’s endemic acroloxid limpets: the role of geography and ecology
Source: BMC Evol Biol. 2016 Dec 15;16:273. doi: 10.1186/s12862-016-0826-6 (PMC5159953; doi:10.1186/s12862-016-0826-6)
Supplement: Additional file 2: Figure S1. — MCC trees for the four BEAST analyses performed (outgroup removed). See Methods and Results for details. Figure S2 MCC trees for the four *BEAST analyses performed (including outgroups). See Methods and Results for details. Figure S3. Combined mitochondrial parsimony network for the two markers 16S rRNA and COI. Position of haplotypes and haplotype groups approximately refer to sampling sites across the lake (compare Fig. 3; yellow: Acroloxus macedonicus; green: non-ribbed A. macedonicus; orange: A. improvisus). Regular numbers refer to DNA voucher numbers; COI haplotype numbers are marked with a hashtag (see Additional file 1: Table S1). Figure S4. The shell of the regular (ribbed) Acroloxus macedonicus (SEM data). A–G, J–K — protoconch (C, F–G — initial plate; E, J–K — sculpture). H–I, L–M — teleoconch (L–M — fragments of ribbed surface). A–B, E, G–H — left view; C–D, I — top view; F — posterior-right view. Scale bars: A– D, F–G, J, L–M = 0.1 mm, E = 0.05 mm, H–I = 1 mm, K = 10 μm. Figure S5. The shell of non-ribbed specimens of Acroloxus macedonicus (SEM data). 1 — first specimen, 2 — second spm. 1A–C, 1E, 2A–C — teleoconch (1E — fragment of smooth surface). 1D, 2D — protoconch. 1A, 2A — left view; 1B, 2B — rear view; 1C–D, 2C–D — top view. Scale bars: 1A– D, 2A–D = 1 mm, 1E = 0.1 mm. Figure S6. The shell of Acroloxus improvisus (SEM data). A–F, I–L — protoconch (E, J, K–L — sculpture; F, I — initial plate). G–H, M — teleoconch (M — fragment of smooth surface). A — posterior-left view; B, F–G — left view; C — right view; D, H — top view; I — right and top view. Scale bars: A–F, I–K, M = 0.1 mm, G–H = 1 mm, L = 10 μm. (PDF 39654 kb) [file 12862_2016_826_MOESM2_ESM.pdf]

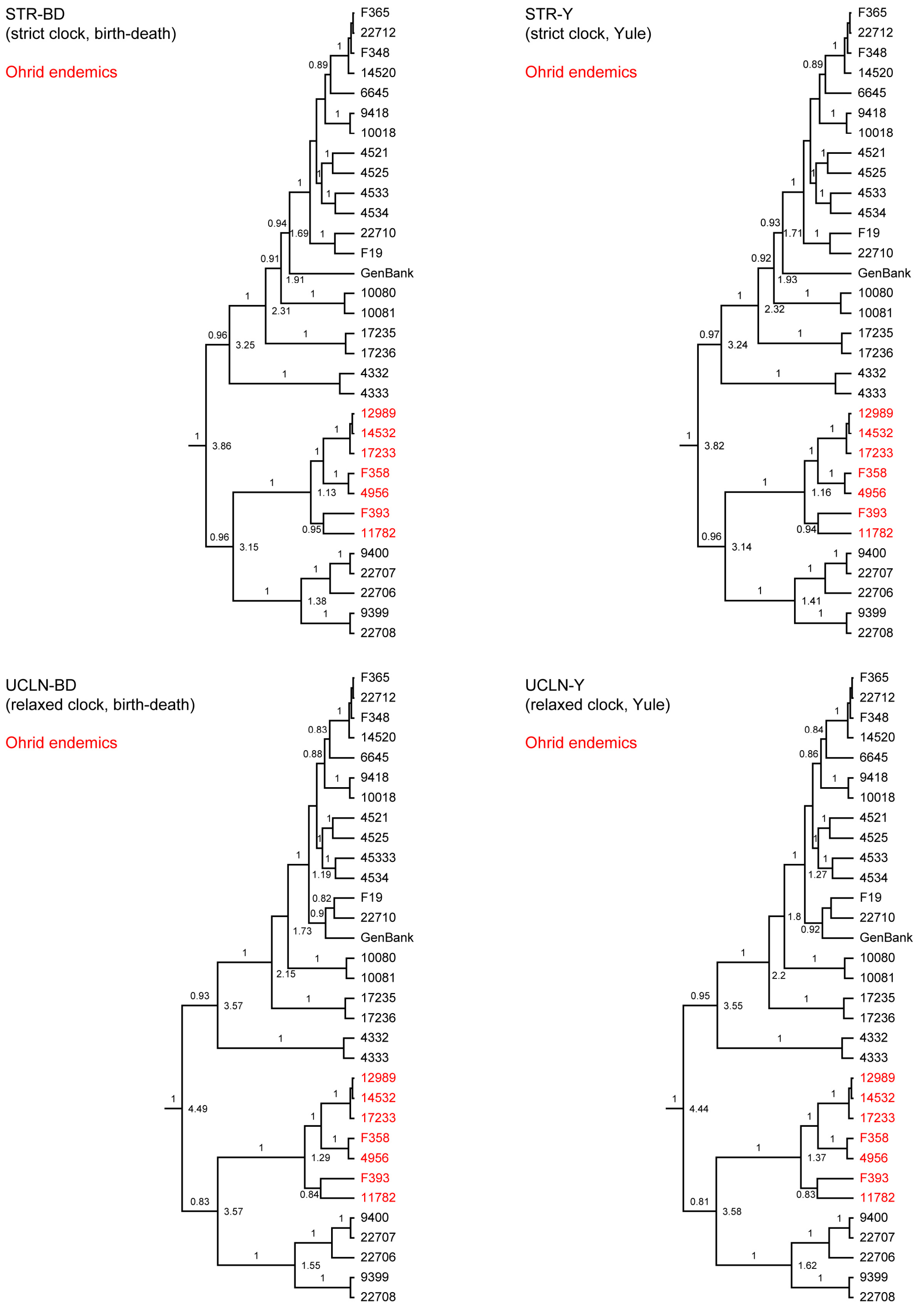

Figure S1 MCC trees for the four BEAST analyses performed (outgroup removed). See Methods and Results for details.

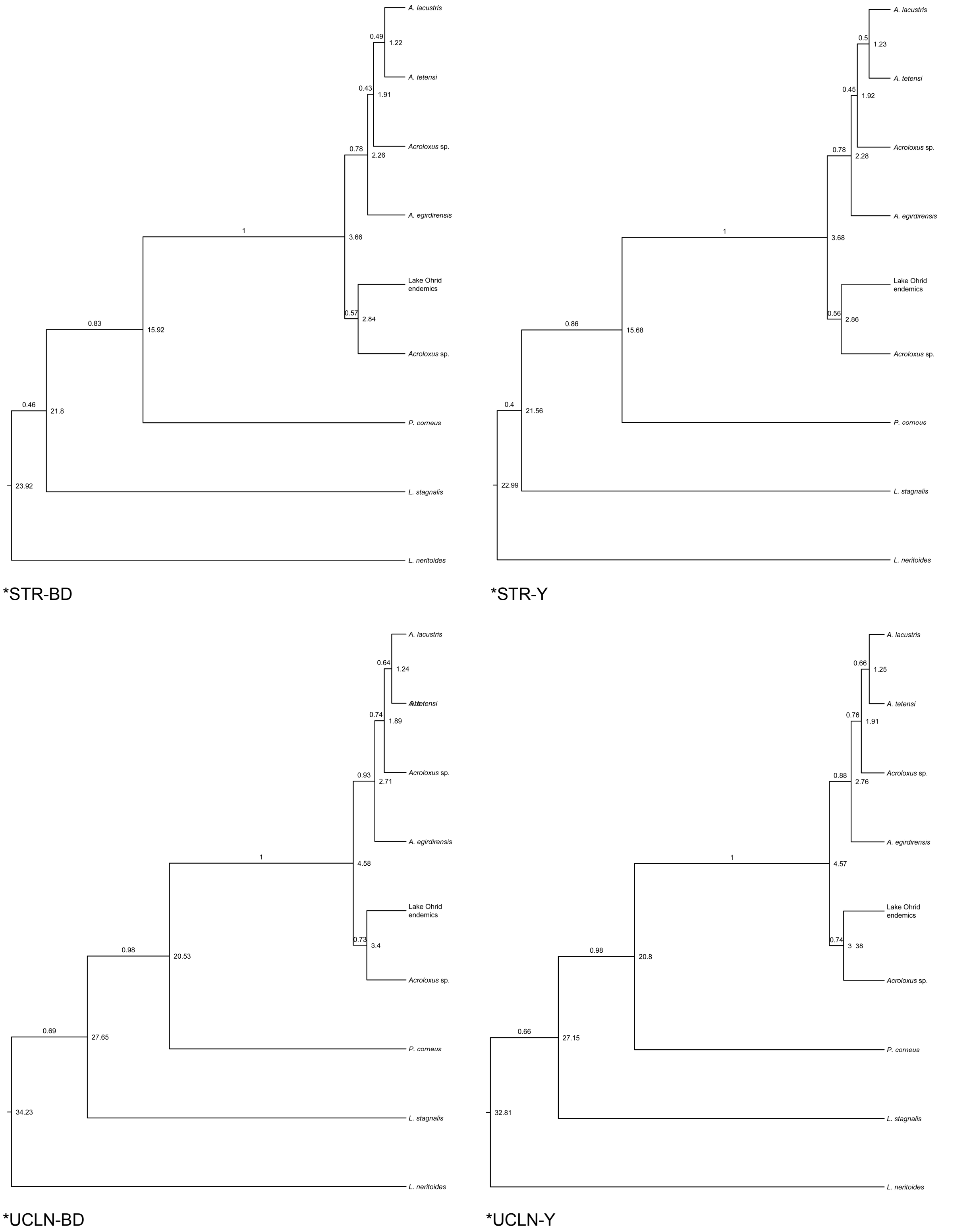

Figure S2 MCC trees for the four *BEAST analyses performed (including outgroups). See Methods and Results for details.

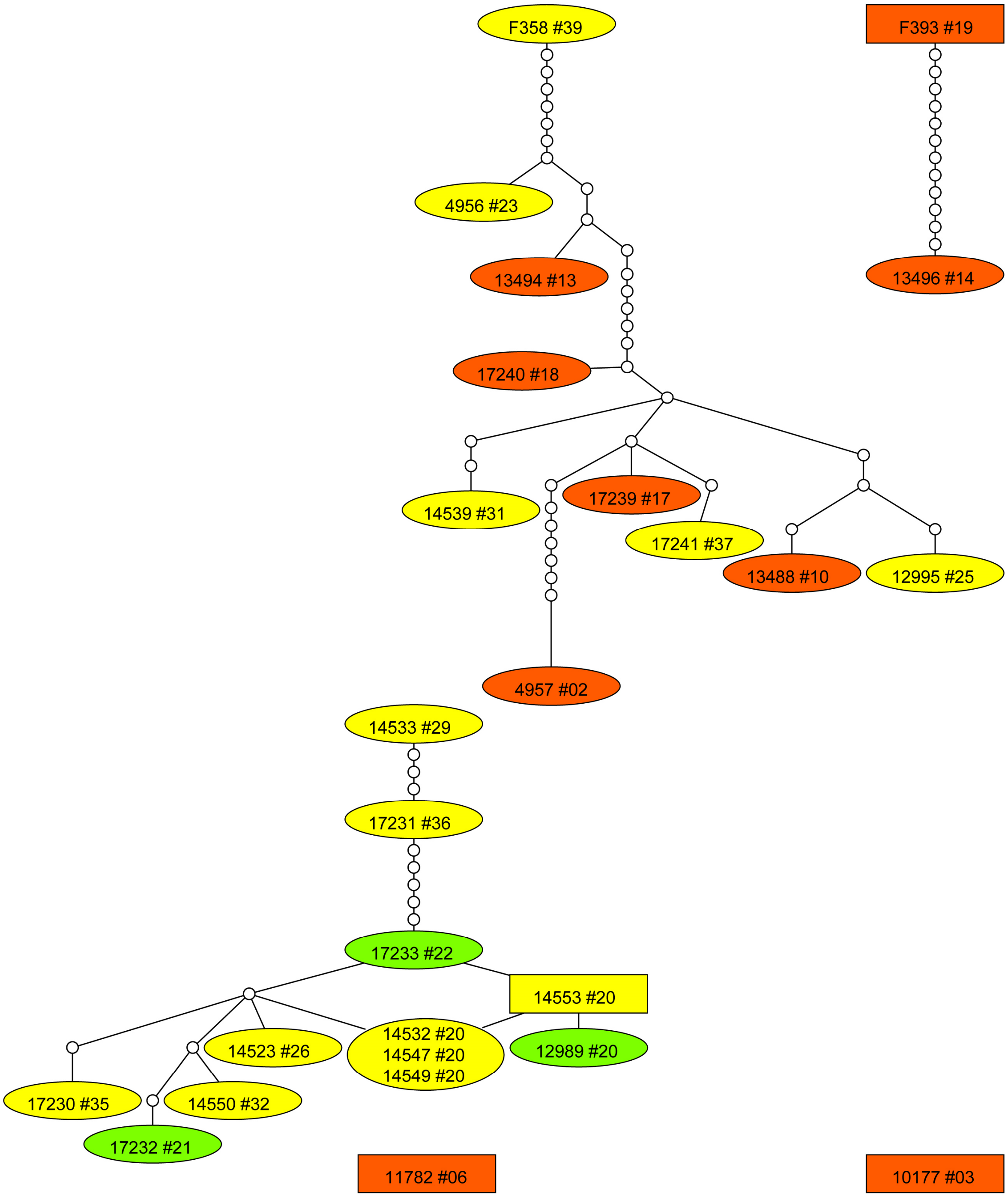

Figure S3 Combined mitochondrial parsimony network for the two markers 16S rRNA and COI. Position of haplotypes and haplotype groups approximately refer to sampling sites across the lake (compare Figure 3; yellow: *Acroloxus macedonicus*; green: non-ribbed *A. macedonicus*; orange: *A. improvisus*). Regular numbers refer to DNA voucher numbers; COI haplotype numbers are marked with a hashtag (see Additional file 1: Table S1).

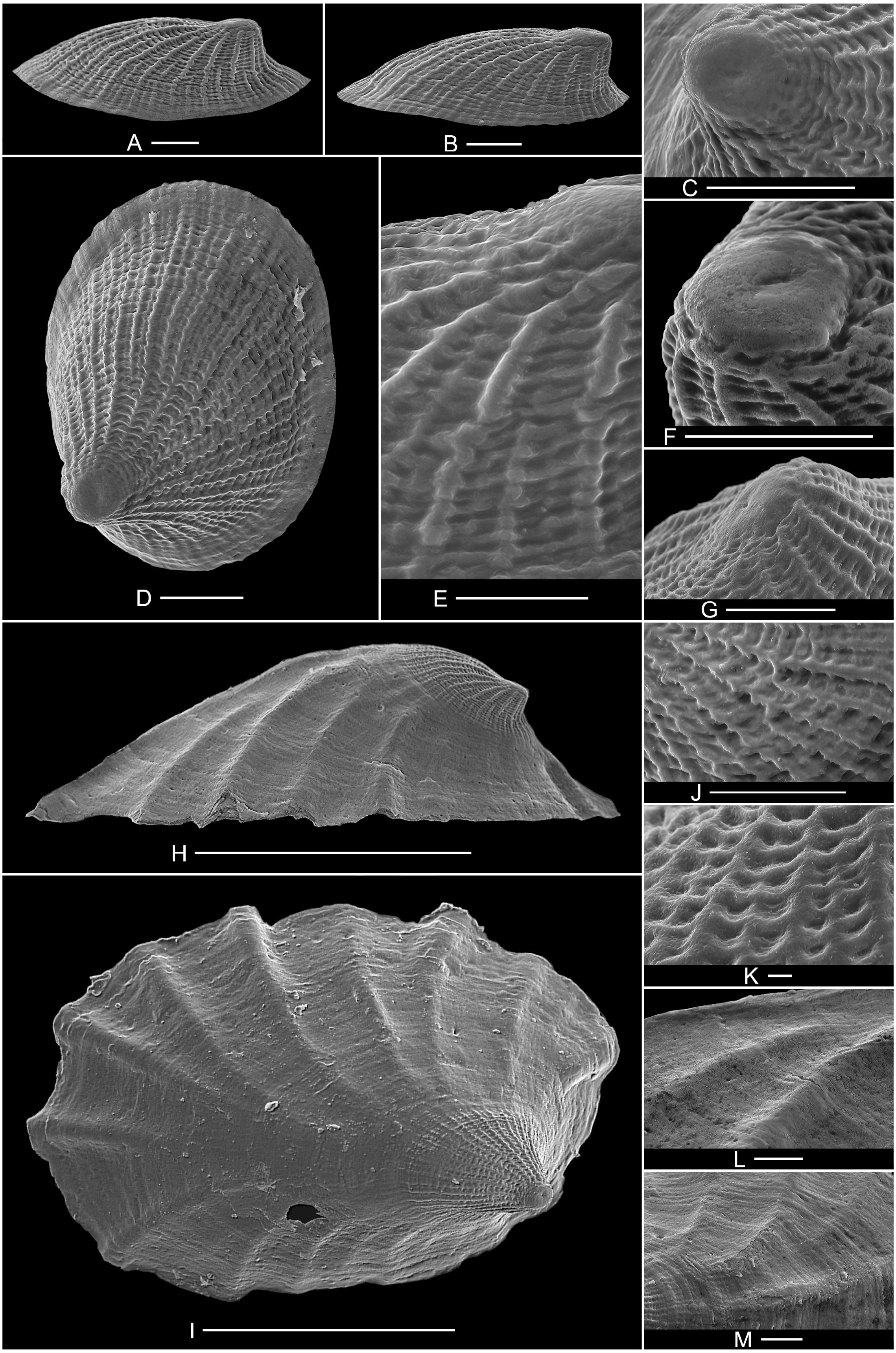

Figure S4 The shell of the regular (ribbed) *Acroloxus macedonicus* (SEM data). A–G, J–K — protoconch (C, F–G — initial plate; E, J–K — sculpture). H–I, L–M — teleoconch (L–M — fragments of ribbed surface). A–B, E, G–H — left view; C–D, I — top view; F — posterior-right view. Scale bars: A–D, F–G, J, L–M = 0.1 mm, E = 0.05 mm, H–I = 1 mm, K = 10 μm.

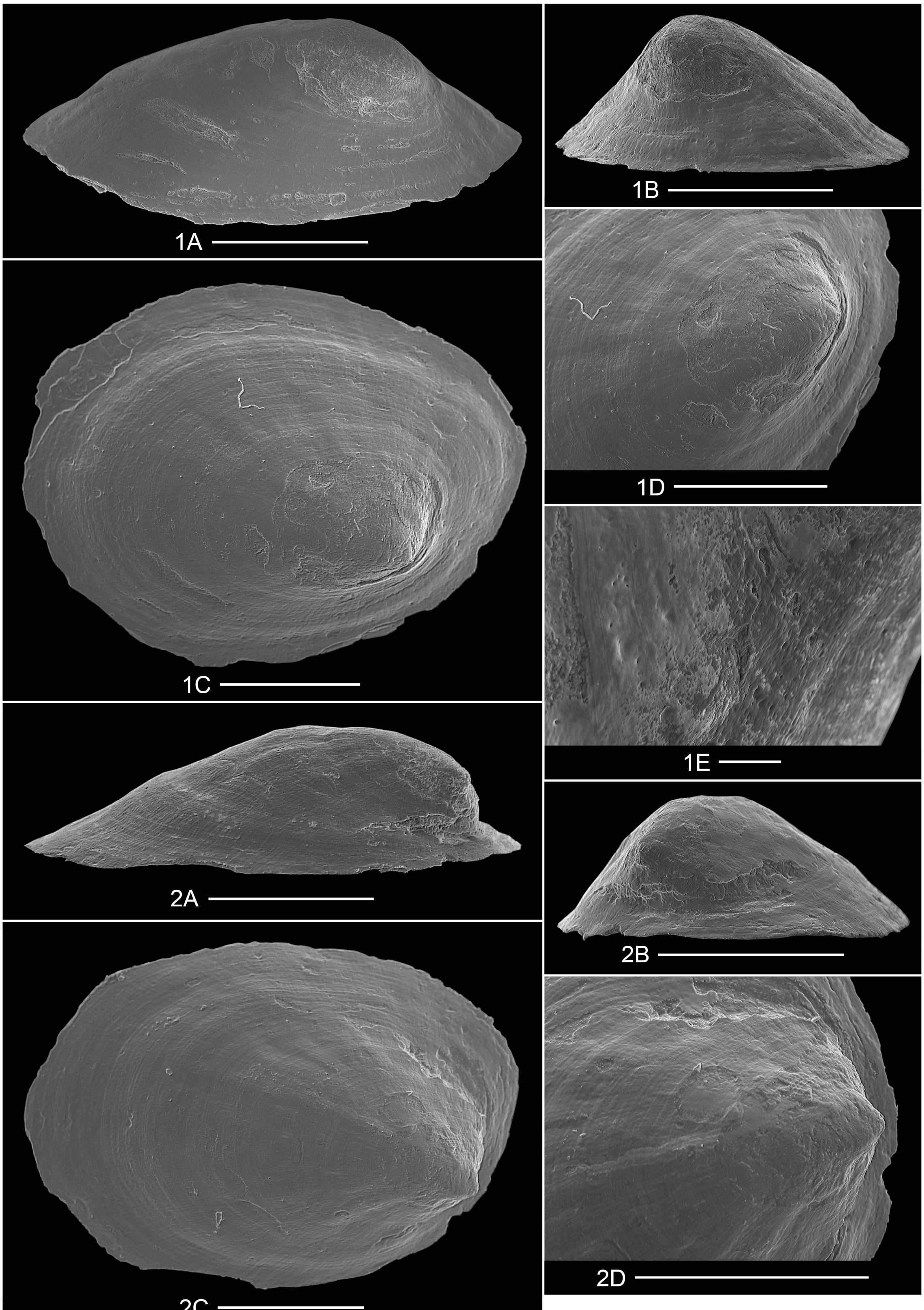

Figure S5 The shell of non-ribbed specimens of *Acroloxus macedonicus* (SEM data). 1 — first specimen, 2 — second spm. 1A–C, 1E, 2A–C — teleoconch (1E — fragment of smooth surface). 1D, 2D — protoconch. 1A, 2A — left view; 1B, 2B — rear view; 1C–D, 2C–D — top view. Scale bars: 1A–D, 2A–D = 1 mm, 1E = 0.1 mm.

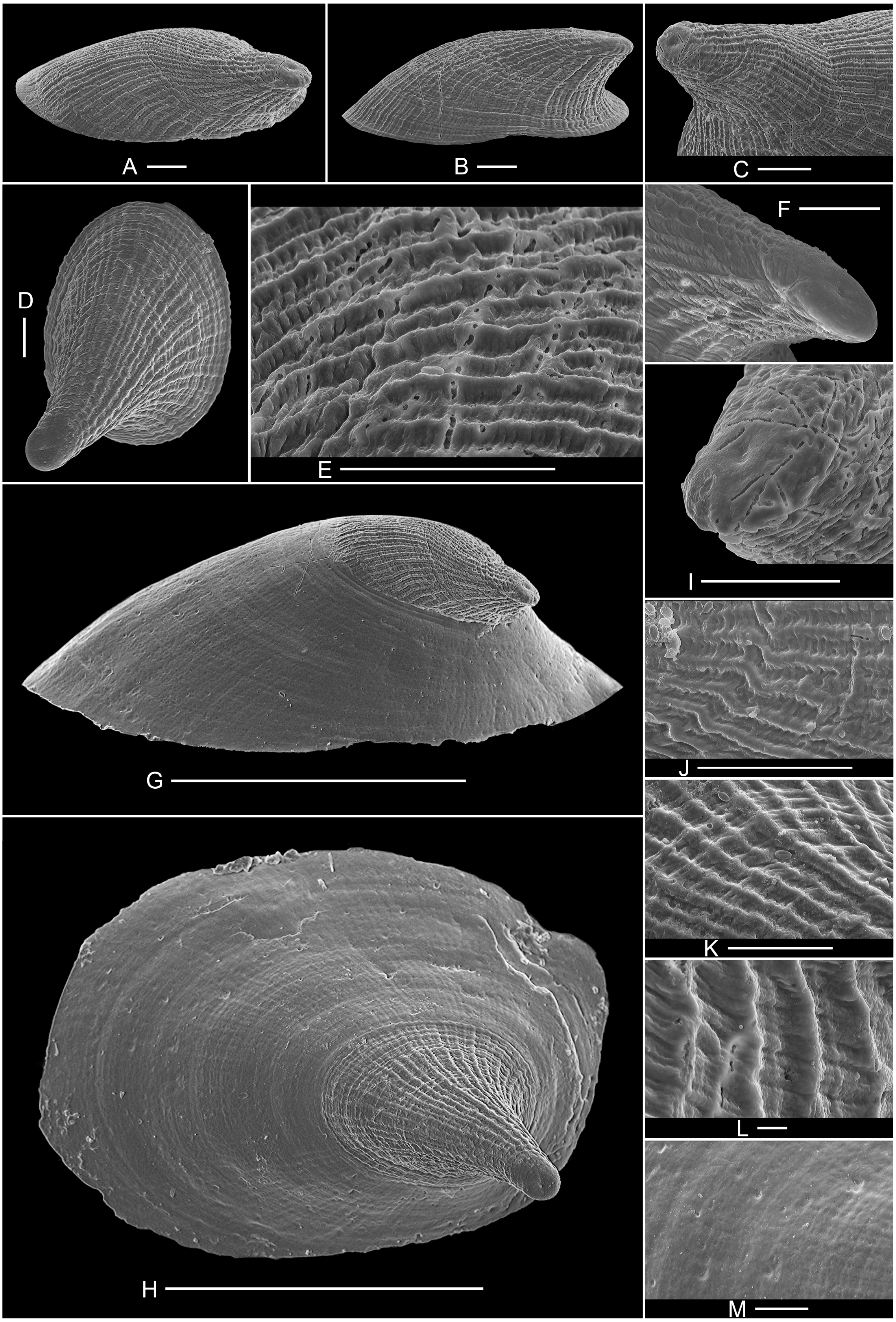

Figure S6 The shell of *Acroloxus improvisus* (SEM data). A–F, I–L — protoconch (E, J, K–L — sculpture; F, I — initial plate). G–H, M — teleoconch (M — fragment of smooth surface). A — posterior-left view; B, F–G — left view; C — right view; D, H — top view; I — right and top view. Scale bars: A–F, I–K, M = 0.1 mm, G–H = 1 mm, L = 10 μm.

Taxonomic remarks on *Acroloxus macedonicus* and *A. improvisus*

Morphological and anatomical differences between the two endemic Lake Ohrid species, *A. macedonicus* Hadžišce, 1959 and *A. improvisus* Polinski, 1929:

Acroloxus macedonicus:

<u>Shell.</u> Apex is blunt. Posterior slope of the protoconch is steep, almost vertical. Tip of the apex does not protrude the posterior edge of the protoconch base. Protoconch with a reticulate microsculpture (visible transverse ridges are present between the radial or longitudinal ridges). Initial plate is always rounded. In some specimens, the sculpture of the teleoconch consists of rounded ridges, causing a wavy appearance of the entire surface; shell is relatively narrow; aperture is oval (W/L = 0.67; W: aperture width, L: aperture length). In other individuals, the radial macrosculpture is missing or almost missing; shell is wide; aperture is rounded (W/L = 0.74–0.75).

<u>Radula.</u> Central tooth is unicuspid.

<u>Anatomy.</u> Pseudobranch is long, reaching down almost to the level of the sole of the foot. Caecum is long and slender.

Acroloxus improvisus:

<u>Shell.</u> Apex is peaked, elongated and looks like a bird beak. Posterior slope of the protoconch is distinctly concave. Tip of the apex protrudes the posterior edge of the protoconch base. Protoconch with an extremely fine longitudinal striation (sometimes twisted). Initial plate oval or oval-rounded. Teleoconch is smooth, convex. Shell is relatively narrow. Aperture is elongate-ovate or oval (W/L = 0.63–0.73).

<u>Radula.</u> Central tooth is asymmetrically bicuspid.

<u>Anatomy.</u> Pseudobranch is short. Caecum is short and thick.

The protoconch (embryonal shell) can be a more useful character for the comparison of freshwater gastropod taxa than the presence/absence of a sculpture on the teleoconch (adult shell; [1]). While the aperture shape and the severity of the sculpture are very variable in acroloxid limpets, the protoconch represents a comparatively conservative morphological character. This also applies to the two endemic species.

The non-ribbed specimens of *Acroloxus macedonicus* (Albanian shore) can be easily distinguished from specimens of *A. improvisus* by the embryonic shell shape. Their protoconchs possess a steep (almost vertical) posterior slope; the tip of the apex does not protrude the posterior edge of the protoconch base (see SEM data above).

References

1. Riedel F. Early ontogenetic shell formation in some freshwater gastropods and taxonomic implications of the protoconch. Limnologica. 1993;23:349–68.
